# Versatile DNA extraction from diverse plant taxa using ionic liquids and magnetic ionic liquids: a methodological breakthrough for enhanced sample utility

**DOI:** 10.1186/s13007-024-01217-z

**Published:** 2024-06-14

**Authors:** Shashini De Silva, Cecilia Cagliero, Morgan R. Gostel, Gabriel Johnson, Jared L. Anderson

**Affiliations:** 1 Department of Chemistry, Iowa State University, Ames, IA 50011 USA; 2 Dipartimento di Scienza e Tecnologia del Farmaco, Università di Torino, Turin, I-10125 Italy; 3 Botanical Research Institute of Texas, Fort Worth, TX 76107-3400 USA; 4 Smithsonian Institution, Suitland, MD 20746 USA

**Keywords:** Plant DNA isolation, Miniaturization, Ionic liquids, Magnetic ionic liquids, Matrix solid phase dispersion, qPCR, Sequencing

## Abstract

**Background:**

There is a growing demand for fast and reliable plant biomolecular analyses. DNA extraction is the major bottleneck in plant nucleic acid-based applications especially due to the complexity of tissues in different plant species. Conventional methods for plant cell lysis and DNA extraction typically require extensive sample preparation processes and large quantities of sample and chemicals, elevated temperatures, and multiple sample transfer steps which pose challenges for high throughput applications.

**Results:**

In a prior investigation, an ionic liquid (IL)-based modified vortex-assisted matrix solid phase dispersion approach was developed using the model plant, *Arabidopsis thaliana* (L.) Heynh. Building upon this foundational study, the present study established a simple, rapid and efficient protocol for DNA extraction from milligram fragments of plant tissue representing a diverse range of taxa from the plant Tree of Life including 13 dicots and 4 monocots. Notably, the approach was successful in extracting DNA from a century old herbarium sample. The isolated DNA was of sufficient quality and quantity for sensitive molecular analyses such as qPCR. Two plant DNA barcoding markers, the plastid *rbcL* and nuclear ribosomal internal transcribed spacer (nrITS) regions were selected for DNA amplification and Sanger sequencing was conducted on PCR products of a representative dicot and monocot species. Successful qPCR amplification of the extracted DNA up to 3 weeks demonstrated that the DNA extracted using this approach remains stable at room temperature for an extended time period prior to downstream analysis.

**Conclusions:**

The method presented here is a rapid and simple approach enabling cell lysis and DNA extraction from 1.5 mg of plant tissue across a broad range of plant taxa. Additional purification prior to DNA amplification is not required due to the compatibility of the extraction solvents with qPCR. The method has tremendous potential for applications in plant biology that require DNA, including barcoding methods for agriculture, conservation, ecology, evolution, and forensics.

**Supplementary Information:**

The online version contains supplementary material available at 10.1186/s13007-024-01217-z.

## Background

Isolation of DNA is a crucial step that forms the foundation of many applications in molecular biology ranging from simple DNA barcoding to comparative genomics [1, 2]. In addition to plant DNA barcoding and genomics, DNA isolation is fundamental to various fields of research including genetically modified organism identification [3, 4] and disease diagnostics [5, 6]. DNA barcoding is a particularly important tool for rapid species identification based on DNA sequences [7]. It involves a series of steps starting with DNA isolation followed by DNA amplification of universal barcode loci, and sequencing. Sequenced barcode loci can then be added to a growing database or used for identification purposes by comparing the sequenced region of DNA to existing barcode reference libraries [8, 9]. DNA barcoding for plants is broadly applied to provide insights into species-level taxonomy and assist in unknown species identification [10] and is useful for many professions and areas of study such as taxonomy, ecology, conservation, forensic science, agriculture, human/animal health and environmental protection [11]. 

It remains a formidable challenge to design a universally applicable DNA extraction method for plants due to the complexity of plant tissues that is compounded by their rigid cell walls and varying levels of polysaccharides, polyphenols, and other secondary (specialized) metabolites among the various phylogenetic lineages. These components, if not adequately eliminated, may hinder the purification process and impede subsequent sensitive DNA molecular analyses [12]. Conventional DNA isolation methods involve surfactants, such as cetyltrimethylammonium bromide (CTAB) [13, 14] or sodium dodecylsulfate (SDS) [14], and heat to facilitate plant cell lysis followed by an additional DNA purification step. DNA purification is often performed by organic solvent-based extraction using phenol and chloroform followed by isopropanol precipitation or solid-phase extraction by silica-based spin columns [5]. These established methods, although effective in giving rise to high yields of DNA, usually involve time-consuming procedures with tedious centrifugation and sample transfer steps which may lead to DNA loss and contamination, particularly when working with very small quantities of precious plant samples. Challenges that arise when dealing with small plant fragments from diverse plant lineages necessitates the development of innovative techniques that yield high-quality DNA suitable for downstream applications such as quantitative PCR (qPCR) and sequencing.

Recently, novel approaches utilizing ionic liquids (ILs) and magnetic ionic liquids (MILs) have been successfully applied for DNA extraction from plant matrices [3, 15]. ILs are organic molten salts possessing melting temperatures at or below 100 ^◦^C. They possess negligible vapor pressures at room temperature, high conductivity, and high thermal and chemical stability [16, 17]. By tuning the cation and anion structures, ILs can be customized to interact with a wide range of important biomolecules [18, 19]. MILs are a subclass of ILs that possess a paramagnetic metal center in the cation and/or anion and often feature similar physico-chemical properties to ILs [20, 21]. The magnetic susceptibility of MILs allows them to be readily manipulated by a magnet in aqueous solutions. The application of ILs and MILs in plant cell lysis, DNA extraction, and DNA preservation have received tremendous attention in recent years. In 2014, Gonzalez García et al. used IL-aqueous buffer systems for the extraction of DNA directly from maize powder followed by a denaturation and filtration step to remove biopolymers [3]. In 2019, Marengo et al. demonstrated the first application of MILs in a magnet assisted-dispersive liquid-liquid microextraction (maDLLME) approach to extract DNA from a plant cell lysate [15]. Plant DNA purified by this approach met the required quality standards for PCR. In 2022, Emaus et al. integrated hydrophobic ILs and MILs into a single step plant cell lysis and DNA extraction method resulting in significantly reduced extraction times. This study demonstrated that plant cells can be simultaneously lysed and DNA extracted by ILs and MILs alone without the need of elevated temperatures or chemical surfactants which can be inhibitory for enzymatic amplification assays [22]. In 2023, De Silva et al. developed a miniaturized vortex-assisted matrix solid phase dispersion approach by integrating an IL and a MIL to extract genomic DNA from plant tissue fragments of the model plant, *Arabidopsis thaliana* (L.) Heynh [23]. DNA extracted by this approach was used for qPCR and could be stored at room temperature in IL- and MIL-cosolvent mixtures.

A miniaturized procedure for DNA isolation is a priority for applications in molecular biology as it will enable DNA to be extracted from smaller sample sizes improving sample utility and reducing sample loss which is crucial when working with limited or precious plant specimens. Miniaturization also reduces the consumption of solvents and sample preparation time while allowing for quicker turnaround in experiments and subsequent analysis. Following successful DNA extraction, DNA barcoding applications require species identification through PCR amplification of a relatively short, standardized genetic loci followed by sequencing. The molecular markers used for DNA barcoding should feature the following aspects: (1) ease of amplification by universal primers, (2) be amenable to bidirectional sequencing and (3) offer maximum discriminatory power in the majority of plant species [10]. A miniaturized platform that enables DNA extraction coupled with PCR amplification using suitable molecular markers and sequencing techniques can be significant in fields such as forensic botany to identify plant taxa from tiny, unknown fragments of plant material found on a suspect or a victim to relate the tissue to a crime scene [24]. Miniaturized procedures can also be useful for DNA extraction from valuable herbarium specimens. Herbaria are curated collections of preserved plant specimens used for scientific investigations [25]. Although herbaria house a large collection of specimens worldwide, only a limited fraction is presently employed for DNA-based research mainly due to the challenges associated with successful DNA extraction and PCR amplification as well as the destructive nature of DNA extraction, which requires the removal of plant fragments from these precious specimens [25]. Access to herbarium DNA is highly beneficial to projects aiming to sample species diversity as herbaria are the largest access points to plant samples with expert-verified species determinations [25]. Therefore, developing a DNA extraction method that can be applied to fresh, preserved and small fragments of plant material from diverse taxa will be beneficial in offering botanical evidence for forensic investigations as well as tapping into the trove of genetic diversity present in historical plant collections from herbaria.

This study addresses the need for a versatile and efficient DNA extraction method tailored for diverse plant lineages that is applicable to small plant fragments. ILs and MILs were integrated into a miniaturized vortex-assisted matrix solid-phase dispersion (VA-MSPD) approach to extract DNA from 1.5 mg plant fragments across 17 plant species belonging to 13 families, including both dicots and monocots, maximizing plant diversity in order to demonstrate the broad utility of this method. DNA extracted by the approach was directly used for qPCR amplification targeting two standard plant DNA barcodes [26], the plastid ribulose-1,5-bisphosphate carboxylase/oxygenase gene (*rbcL*) and a portion of the nuclear ribosomal internal transcribed spacer (nrITS). Additional purification steps were unnecessary due to the compatibility of the solvents with qPCR. Furthermore, the quality of DNA extracted by the approach for Sanger sequencing was explored for a monocot and dicot species. The innovative features of the method enabled it to yield DNA of suitable quality for successful DNA amplification of both *rbcL* and ITS markers, as well as successful sequencing results for a century old herbarium specimen. After storage for a period of 21 days, DNA preserved in the IL- and MIL-cosolvent mixtures demonstrated successful qPCR amplification for the majority of tested plant species. The simplicity and broad applicability of the method positions it as a valuable resource for researchers who require DNA extractions from diverse plant lineages.

## Results

### DNA extraction by IL-based VA-MSPD and amplification of ITS

**Fig. 1:**
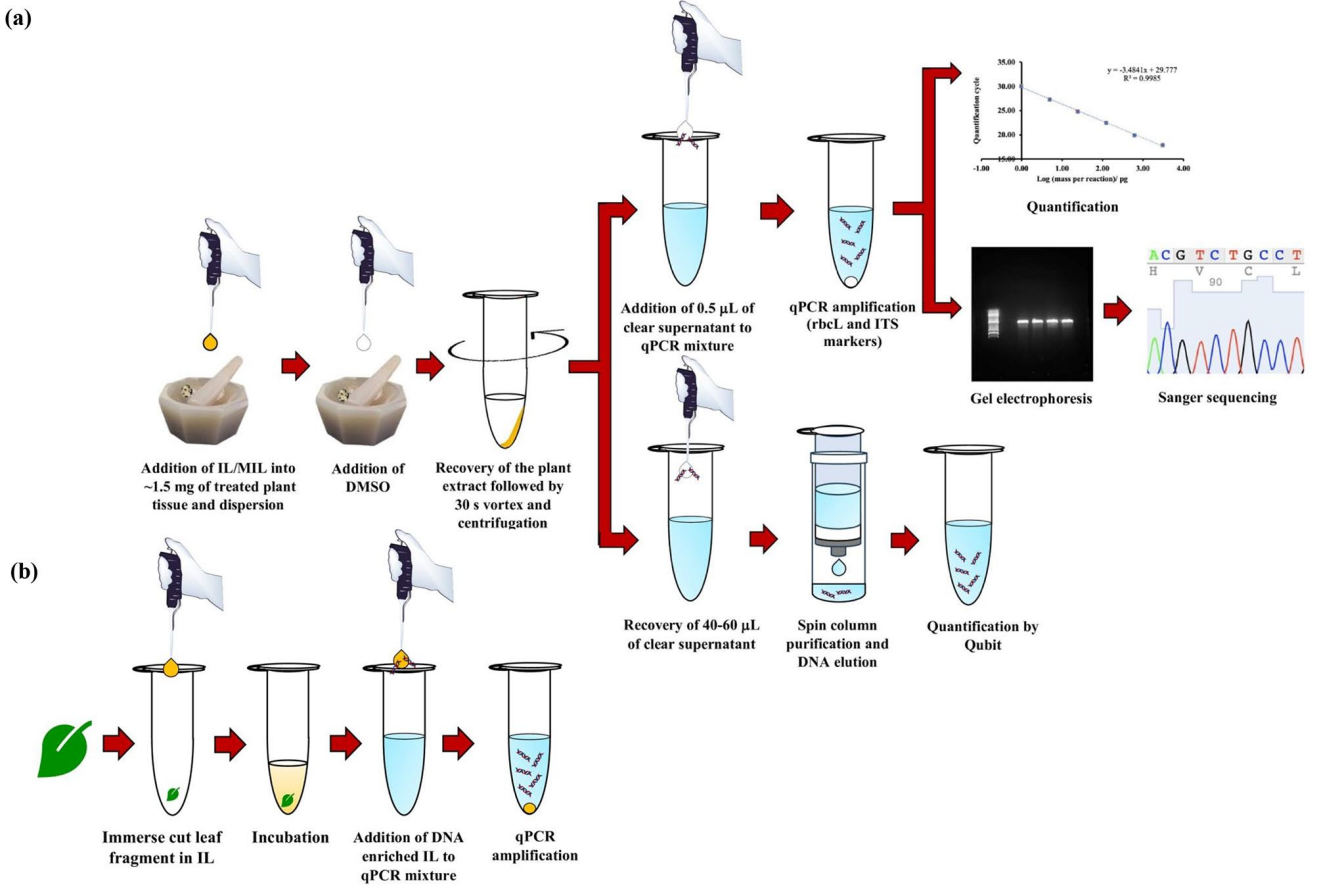
Schematic diagram illustrating the **(a)** IL- based VA-MSPD approach and **(b)** direct IL- based extraction for the isolation of DNA from 1.5 mg of plant tissue using 15 µL of IL / MIL. (adapted from [23])

The IL-based VA-MSPD procedure employed in this study, along with its application, is illustrated in Fig. 1a. Many plant systematists commonly misinterpret the notion that subjecting leaf tissue to ethanol results in the degradation of DNA. Preservation of plant tissues in ethanol differs from spraying ethanol to prevent fungal growth in plant specimens [27]. During ethanol spraying, the plant tissue is only superficially covered with a low concentration of ethanol preserving only the gross morphology of the plant tissue causing the internal tissues to deteriorate and DNA to degrade [28]. Numerous studies have shown the utilization of ethanol pretreatment for successful extraction of DNA from plant tissues [28–30]. In this study, pretreatment of freshly collected plant tissues was carried out in ethanol to preserve the tissue and remove chlorophyll and secondary metabolites [23]. However, it was found that the leaching of plant pigments such as chlorophyll, was not complete for some plant tissues after 12 h of pretreatment. Therefore, fresh solvent was added, and sample pretreatment was carried out for an additional 3 h. The mass loss upon sample pretreatment ranged from 58.32 ± 1.90% to 94.56 ± 0.17%, as shown in Fig. S1. Control experiments, which included air-dried plant tissue without any pretreatment and tissues dehydrated in ethanol for 0.5 h and 12 h, resulted in successful qPCR amplification for the tested samples, and no significant differences in DNA yields were observed across the different types of tissue (Fig. S2).

**Fig. 2:**
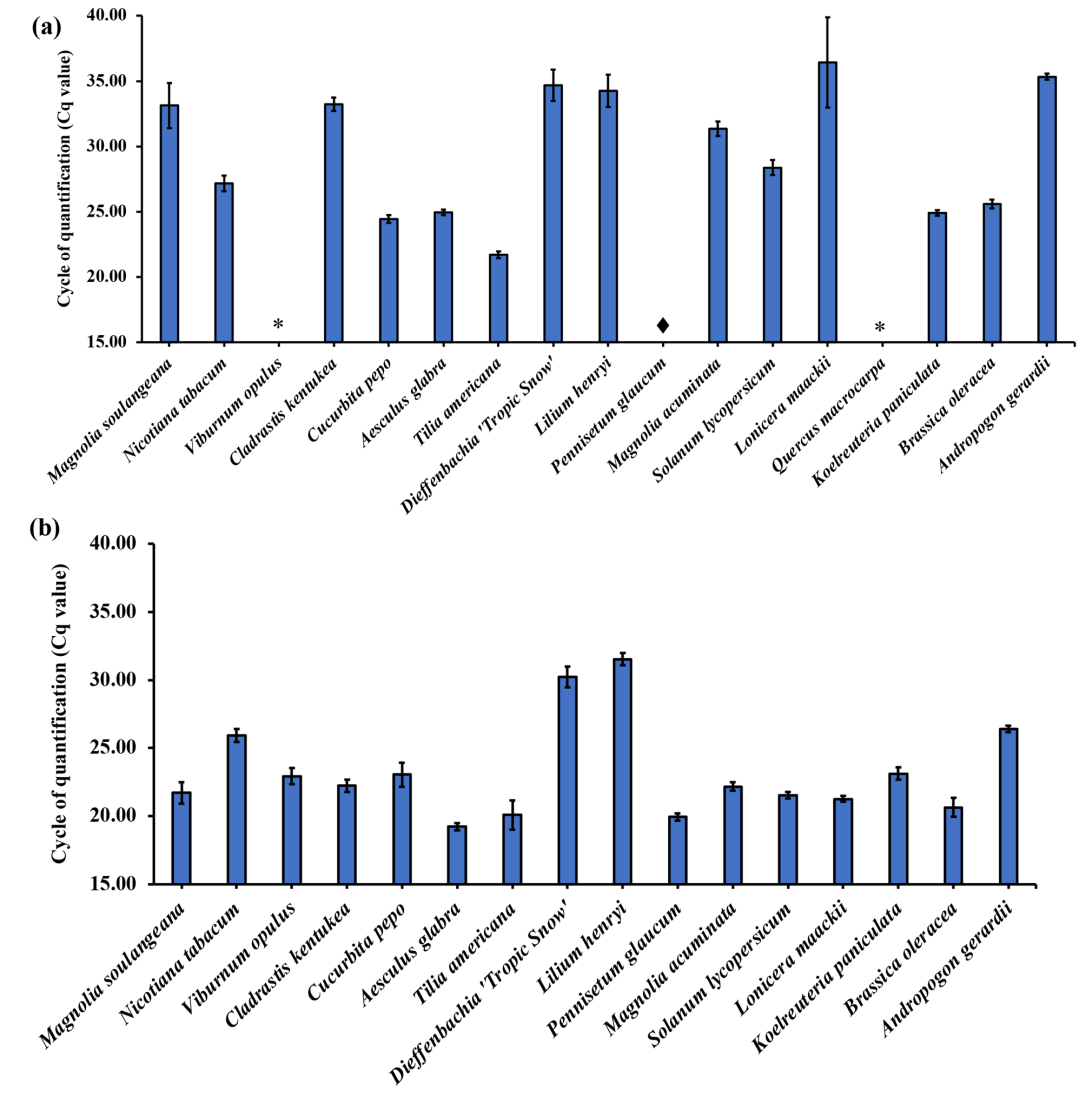
Cq values as a measure of amplification success for **(a)** the ITS marker and **(b)**  *rbcL* marker derived from qPCR amplification of plant DNA extracted by the IL-VA-MSPD procedure employing 1.5 mg of treated plant tissue and 15 µL of [P_6,6,6,14_^+^] [NTf_2_^-^] IL. Extractions were carried out in triplicate. (Cq > 30 is considered as delayed amplification) Note: *Complete inhibition of PCR was observed of *Quercus macrocarpa* therefore *rbcL* amplification was not carried out. ♦ A Cq value was not determined due to delayed amplification

Extractions were performed using 1.5 mg of ethanol treated plant tissue from 17 plant species and qPCR amplification was carried out using universal ITS3 and ITS4 primers to evaluate the suitability of extracted DNA for qPCR. Successful qPCR amplification of ITS was achieved for *Aesculus glabra* Willd., *Tilia americana* L., *Koelreuteria paniculata* Laxm., *Cucurbita pepo* L., *Solanum lycopersicum* L., *Brassica oleracea* L. and *Nicotiana tabacum* L., as shown in Fig. 2a. However, delayed amplification (Cq > 30) was observed for *Magnolia soulangeana* Soul.-Bod., *Lonicera maackii* (Rupr.) Herder, *Cladrastis kentukea* (Dum. Cours.) Rudd, *Dieffenbachia* ‘Tropic Snow’, *Lilium henryi* Baker, *Magnolia acuminata* (L.) L., *Pennisetum glaucum* R. Br. and *Andropogon gerardii* Vitman and complete inhibition was observed for *Viburnum opulus* L. and *Quercus macrocarpa* Michx. Plant species that exhibited delayed qPCR amplification for the ITS region demonstrated either inconsistent or no melt peaks (as shown in Figs S3, S4, S5, S6 and S7), presented non-specific bands on agarose gel (as shown in Fig. S9) or demonstrated both phenomena.

**Fig. 3:**
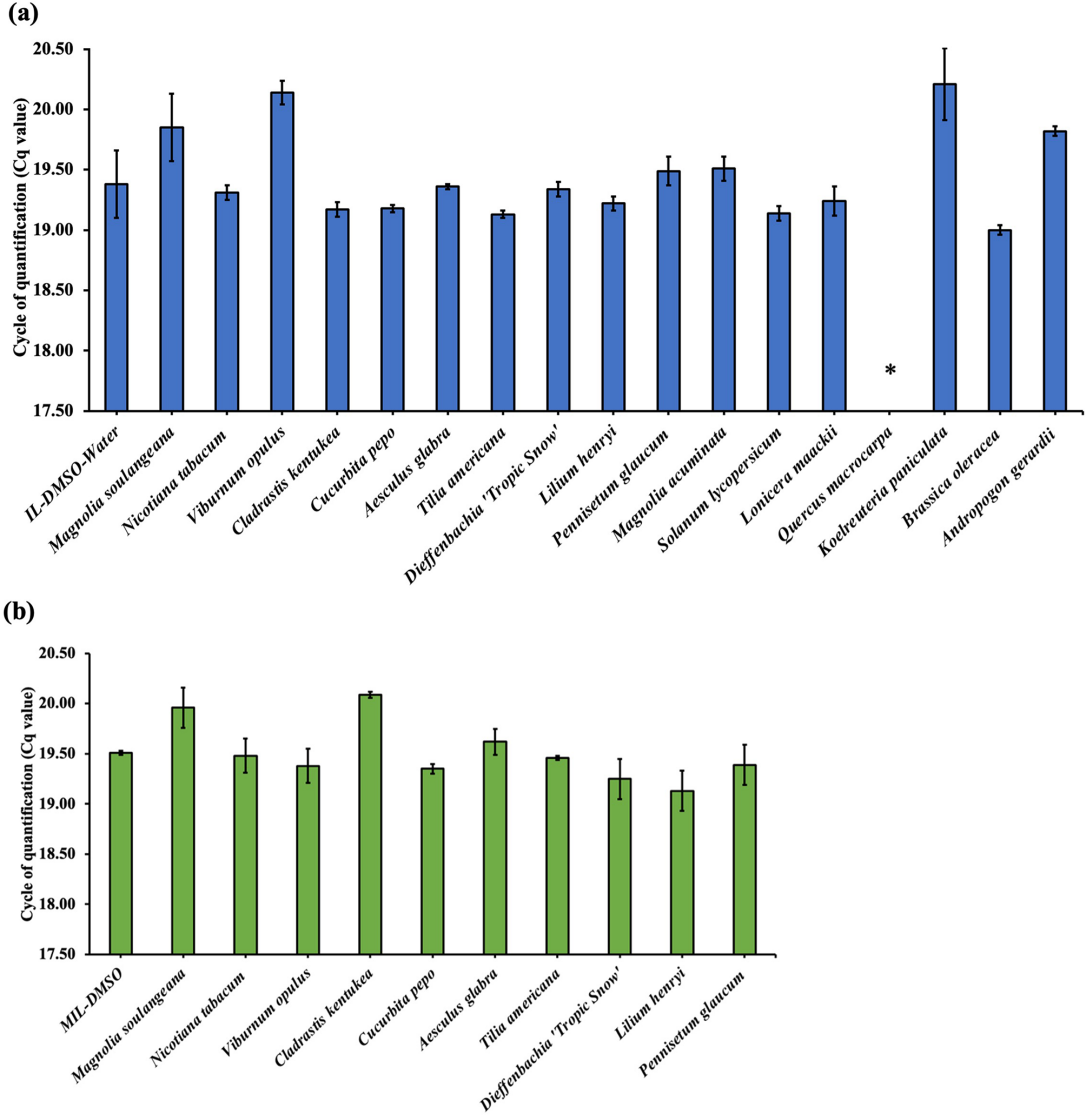
Effect of the plant matrix on the amplification of non-target 98 bp BRAF DNA template. A volume of 1 µL of 10.2 fg/µL non-target 98 bp DNA template (BRAF) was spiked into the qPCR assay and amplified in the presence of **(a)** 0.5 µL of the IL-DMSO-Water extract and **(b)** 0.5 µL of the MIL-DMSO extract containing plant DNA. All experiments were carried out in triplicate. Note: *Complete inhibition of PCR was observed

To test if any component in the extract affects qPCR amplification, 1 µL of 10.2 fg/µL non-target 98 bp DNA template (BRAF) was spiked into the qPCR assay and amplified with 0.5 µL of the plant extract. The BRAF gene, located on chromosome 7 in the human genome, encodes for B-raf protein and is well known for its role in human cancer [31]. It is not commonly found in plants and served as a control DNA sequence. BRAF DNA amplified successfully in the presence of plant extract with Cq values having standard deviations of ± 0.5 cycles compared to that of the control. *Q. macrocarpa* was an exception and exhibited complete inhibition as did *V. opulus* and *K. paniculata*, which demonstrated slightly delayed amplification with Cq values of 20.14±0.10 and 20.21±0.30 respectively, as shown in Fig. 3a.

The IL-based VA-MSPD approach for plant DNA extraction involves dispersing the homogenized plant material with the IL to facilitate plant cell lysis and DNA extraction, followed by addition of cosolvent to enable the recovery of the mixture (Fig. 1a). Since the extraction step involves grinding the plant tissue with the extraction solvent, it is possible to achieve higher DNA co-extraction of qPCR inhibitors compared to that from a static extraction. Therefore, direct IL-based extraction was performed for four of the challenging plants that demonstrated delayed or no ITS amplification, such as *M. soulangeana, V. opulus, Q. macrocarpa* and *L. maackii* by placing 15 µL of the IL directly onto 1.5 mg of plant tissue, thereby facilitating the static extraction in an effort to limit co-extraction of impurities (Fig. 1b). No improvement in amplification was observed from static extractions, indicating that the co-extraction of inhibitors is not the only reason for delayed amplification.

It was hypothesized that the observed delayed amplification for most of the plants was due to the following two reasons: (1) extraction method was not ideal for some of the plants chosen, and (2) low qPCR amplification success for ITS region of the plant. To test the first hypothesis, an alternative plant species from the same family as *M. soulangeana* was tested. *Magnolia acuminata* (L.) L., which belongs to the family Magnoliaceae, was subjected to the same extraction method and the ITS region amplified. However, no improvement in ITS amplification was observed even for the alternative plant (Cq > 30). Therefore, an additional marker was tested.

### DNA extraction by IL-based VA-MSPD and amplification of *rbcL* barcoding region

To evaluate the qPCR amplification success of the *rbcL* marker in this study, assay optimization was performed for genomic DNA of *A. thaliana* using universal *rbcLa* primers. A qPCR efficiency of 94.36% was achieved for reactions containing IL-DMSO-water mixtures. IL-based extraction was then performed for *M. soulangeana* and *rbcL* region amplified as it is among the more difficult plants to achieve qPCR success, as indicated by greatly delayed amplification for ITS. Improved amplification for *rbcL* was achieved compared to ITS as shown by Fig. S3. Additionally, defined melt peaks for the *rbcL* amplicon were observed for *M. soulangeana* compared to its ITS amplicon (Fig. S3). Similarly, *V. opulus* and *L. maackii* (which also did not show successful amplification with ITS) demonstrated amplification success with *rbcL* with defined melt peaks as shown by Figs. S4 and S5, respectively. Among the monocots tested, *Dieffenbachia* ‘Tropic Snow’ and *L. henryi* exhibited delayed amplification for both *rbcL* (Cq > 30) and ITS (Cq > 30) whereas *P. glaucum* and *A. gerardii* produced successful amplification for *rbcL* but not for ITS (shown by Figs. S6 and S7). Similarly, *rbcL* amplification was carried out for the remaining plant extracts and successful amplification was achieved for the majority of plants (Fig. 2b). All *rbcL* PCR products produced single bands in agarose gels, as shown by Figs. S8 and S9.

### DNA extraction by MIL-based VA-MSPD and amplification of *rbcL* and ITS

As the [P_66614_^+^][Ni(hfacac)_3_^−^ ] MIL demonstrated greater DNA extraction capability as well as stability for *A. thaliana* based on a previous study [23], the MIL was also explored as an extraction solvent for 10 plant species that did not have duplicated higher order taxa (Table 1). Successful qPCR amplification was achieved for *rbcL* for all tested plant species, except for *P. glaucum* which did not show amplification and *L. henryi* which showed delayed amplification (Cq > 30), as seen in Fig. S10a. All *rbcL* PCR products produced single bands in agarose gels (Fig. S9). ITS amplification was carried out on 5 of the tested species yielding successful amplification while the remaining gave rise to delayed amplification (Fig. S10b).

**Table 1:** List of plant species tested and their corresponding classification

Plant species	Family	Order	Super class/clade
1. *Magnolia soulangeana* Soul.-Bod.	Magnoliaceae	Magnoliales	Magnoliids
2. *Nicotiana tabacum* L.	Solanaceae	Solanales	Asterid I
3. *Viburnum opulus* L.	Adoxaceae	Dipsacales	Asterid II
4. *Cladrastis kentukea* (Dum. Cours.) Rudd	Fabaceae	Fabales	Rosid I / Fabidae
5. *Cucurbita pepo* L.	Cucurbitaceae	Cucurbitales	Rosid I / Fabidae
6. *Aesculus glabra* Willd.	Sapindaceae	Sapindales	Rosid II/Malvidae
7. *Tilia americana* L.	Malvaceae	Malvales	Rosid II/Malvidae
8. *Dieffenbachia* ‘Tropic Snow’	Araceae	Arecales	Commelinids
9. *Lilium henryi* Baker	Liliaceae	Liliales	Commelinids
10. *Pennisetum glaucum* R. Br.	Poaceae	Poales	Commelinids
11. *Magnolia acuminata* (L.) L.*	Magnoliaceae	Magnoliales	Magnoliids
12. *Solanum lycopersicum* L.*	Solanaceae	Solanales	Asterid I
13. *Lonicera maackii* (Rupr.) Herder*	Caprifoliaceae	Dipsacales	Asterid II
14. *Quercus macrocarpa* Michx. *	Fagaceae	Fagales	Rosid I / Fabidae
15. *Koelreuteria paniculata* Laxm. *	Sapindaceae	Sapindales	Rosid II/Malvidae
16. *Brassica oleracea* L.*	Brassicaceae	Brassicales	Rosid II/Malvidae
17. *Andropogon gerardii* Vitman *	Poaceae	Poales	Commelinids

* Only IL-based extraction was carried out on these plant tissues

Tests were performed by spiking 1 µL of 10.2 fg/µL BRAF DNA template into the qPCR assay followed by amplification in the presence of 0.5 µL of the MIL-DMSO plant extract. Successful qPCR amplification of the target DNA was achieved for all reactions possessing plant DNA, as shown in Fig. 3b. Reactions containing the control DNA template with 0.5 µL of the plant extract resulted in Cq values having standard deviations of ± 0.5 cycles compared to that of the control, except for C. kentuckea which demonstrated slightly delayed amplification with Cq values of 20.09±0.03.

### Stability of extracted DNA upon storage

**Fig. 4:**
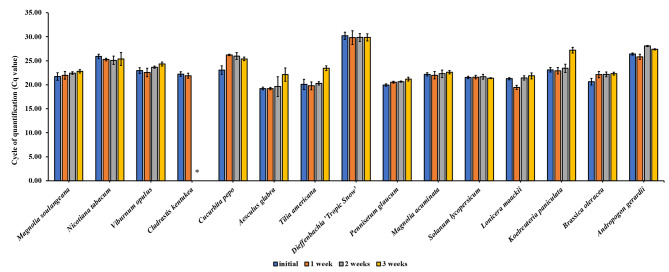
Stability of extracted DNA over time from 1.5 mg of treated plant tissue using 15 µL of [P_6,6,6,14_^+^] [NTf_2_^−^] IL. The MSPD procedure was used in the extraction and DNA was stored in IL-DMSO-water mixture at room temperature. Stability evaluated in terms of Cq values as a measure of amplification success for the *rbcL* marker. All experiments were conducted in triplicate. Note: *A Cq value was not determined after 2 weeks due to diminished fluorescence in the amplification curves

**Fig. 5:**
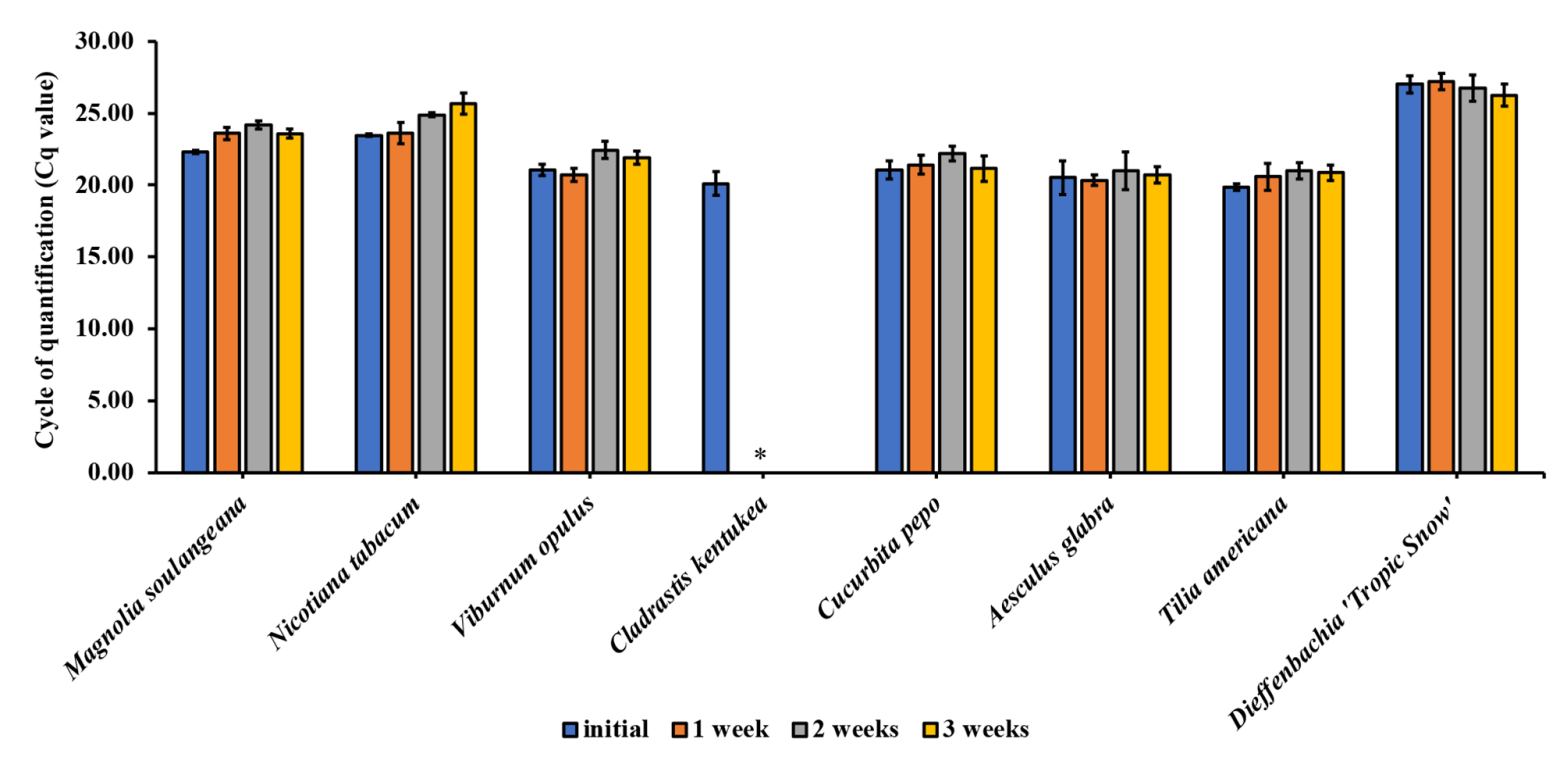
Stability of extracted DNA over time from 1.5 mg of treated plant tissue using 15 µL of [P_6,6,6,14_^+^] [Ni(hfacac)_3_^−^] MIL. The MSPD procedure was used in the extraction and DNA was stored in Ni MIL-DMSO mixture at room temperature. Stability evaluated in terms of Cq values as a measure of amplification success for the *rbcL* marker. All experiments were conducted in triplicate. Note: *A Cq value was not determined after 1 week due to diminished fluorescence in the amplification curves. (Stability tests were not performed for *Lilium henryi* and *Pennisetum glaucum* due to the delayed or no amplification in the initial experiments)

The stability of extracted plant DNA in the IL-DMSO-water and Ni MIL-DMSO extracts upon storage was also investigated as shown in Fig. 4 and 5, respectively. Plant extracts were stored at room temperature for 3 weeks and qPCR measurements were performed every week by amplifying the *rbcL* region to evaluate the length of time that template DNA can be amplified. Successful qPCR amplification was achieved for 3 weeks for the majority of plants. C. kentukea demonstrated decreased fluorescence intensities in the amplification curves after a period of 2 weeks for IL-DMSO-water extracts and 1 week for the MIL-DMSO extracts.

### DNA extraction from herbarium vouchers

Herbarium vouchers are a valuable source of information for various scientific disciplines such as genetic, ecological, taxonomic and/or environmental research [32]. However, DNA extraction from historical specimens and subsequent use of those extractions for downstream amplification or sequencing purposes poses a challenge as the DNA is often highly degraded and fragmented [33]. Given the success of the established method in efficiently extracting and amplifying DNA from small amounts of plant samples across a diverse range of taxa, its utility was expanded to test extraction from herbarium samples as well. *C. pepo* is among the plant specimens that exhibited successful amplification of both the *rbcL* and ITS markers using IL and MIL. Therefore, a herbarium sample of the same species, dating back to 1919, was chosen for the study. The IL-based VA-MSPD approach was capable of extracting DNA from the herbarium specimen over a century old, enabling successful qPCR amplification for both *rbcL* and ITS markers (Figs. S11a and S11b). The mass of DNA extracted from 1.5 mg of herbarium specimen was found to be 9.35±1.84 ng per mg of plant tissue.

### Developing qPCR assays for *rbcL* and ITS for DNA quantification

Among the conventional methods of DNA quantification are UV-spectroscopy and fluorometry, which provide a measure of the total DNA present in the sample irrespective of its origin. These methods are not able to differentiate between DNA from botanical samples and that from other sources such as bacteria, fungus, or animal. Although they are useful in certain applications, they are unable to quantify DNA when present in small quantities due to the interference of background noise necessitating a substantial amount of DNA template in order to give rise to a detectable signal. qPCR is advantageous for DNA quantification as very low amounts of DNA template are sufficient for amplification. To quantify DNA by qPCR, standard curves are required. As the input DNA for qPCR is genomic DNA, calibration curves were constructed using genomic DNA as the template. A series of five-fold dilutions of *A. thaliana* genomic DNA covering a concentration range of 1.82 ng/µL to 0.58 pg/µL were prepared and the *rbcL* region was amplified in the presence of 0.5 µL IL-DMSO-water and MIL-DMSO mixtures in an assay with universal *rbcLa* primers. Melt curve analysis revealed a single melt peak indicative of amplification specificity. qPCR efficiencies of 94.36% and 104.03% were achieved for *rbcL* for reactions containing IL-DMSO-water mixtures and MIL-DMSO mixtures, respectively. qPCR efficiencies of 96.40% and 97.14% have been previously reported for ITS amplification of *A. thaliana* genomic DNA for the same mixtures [23]. 

Standard curves were also constructed for *C. pepo, M. soulangeana, Dieffenbachia* ‘Tropic Snow’ targeting both ITS and *rbcL* regions and the qPCR efficiencies, coefficient of determination and the slopes of the standard curves are summarized in Table 2. Selection of three representative plant species for performing standard curves was based on the amplification success of the plant species for *rbcL* and ITS where *C. pepo* showed successful amplification for both *rbcL* and ITS, *M. soulangeana* showed successful amplification for only *rbcL* but not ITS and *Dieffenbachia* ‘Tropic Snow’ (a monocot species) showed delayed amplification for both *rbcL* and ITS. qPCR efficiencies associated with IL-DMSO-water and MIL-DMSO mixtures for rbcL and ITS markers were found to be within 90–105% for *A. thaliana* and *C. pepo.* qPCR efficiency associated with IL-DMSO-water for ITS marker of *M. soulangeana* was 99.34% and MIL-DMSO mixture was above 105%. However, clearly defined melt peaks were not observed for the ITS amplicon for *M. soulangeana* for both mixtures. The qPCR efficiency for MIL-DMSO mixtures using the *rbcL* marker in *C. pepo* was 80.61%, which is below the accepted qPCR efficiency range for reliable quantification. Nonetheless, the assay was target specific as a single melt peak was observed for the entire concentration range. The inefficiency could be attributed to interference of the MIL-DMSO mixture with the enzymatic assay. qPCR assays associated with IL-DMSO-water and MIL-DMSO mixtures using *rbcL* and ITS markers of *Dieffenbachia* ‘Tropic Snow’ were all inefficient (data not shown) and this is likely due to non-specific amplification as a single melt peak was not observed across the different concentrations tested. Studies have shown that PCR efficiency varies across different barcoding markers and species and that Cq values can be used for assessing PCR success [34]. With the use of efficient standard curves, the mass of DNA extracted by the [P_66614_^+^][NTf_2_^−^ ] IL was quantified as 7.71 ± 4.81 ng/mg of plant tissue and 23.48 ± 1.57 ng/mg of plant tissue for *C. pepo* and *M. soulangeana*, respectively. The mass of DNA extracted by the [P_66614_^+^][Ni(hfacac)_3_^−^] MIL was 33.85 ± 2.31 ng/mg of plant tissue for *M. soulengeana.*

**Table 2:** qPCR efficiencies, coefficient of determination (R^2^ values) and slopes of calibration curves for qPCR assays using *rbcL* and ITS markers containing (a) 0.5 µL of 1:2:1 (v/v/v) mixture of [P_6,6,6,14_^+^][NTf_2_^-^] IL, DMSO and water and (b) 0.5 µL of 1:4 (v/v) mixture of [P_6,6,6,14_^+^][Ni(hfacac)_3_^-^] MIL and DMSO for *A. thaliana, C. pepo* and *M. soulangeana* genomic DNA.

**(a)**						
**Plant species**	**rbcL_IL-DMSO-water**			**ITS_IL-DMSO-water**		
	**Efficiency**	**Slope**	*R*^2^ value	**Efficiency**	**Slope**	*R*^2^ value
*A. thaliana*	94.36%	-3.4649	0.9992	96.40%*	-3.4113*	0.9993*
*C. pepo*	93.65%	-3.4841	0.9985	93.40%	-3.4909	0.9988
*M. soulangeana*	93.88%	-3.4778	0.9980	99.34%	-3.3378	0.9971
**(b)**						
**Plant species**	**rbcL_MIL-DMSO**			**ITS_MIL-DMSO**		
	**Efficiency**	**Slope**	*R*^2^ value	**Efficiency**	**Slope**	*R*^2^ value
*A. thaliana*	104.03%	-3.2289	0.9954	97.14%*	-3.3922*	0.9997*
*C. pepo*.	80.61%	-3.895	0.9991	91.39%	-3.5471	0.9953
*M. soulangeana*	90.45%	-3.5743	0.9970	134.2%	-2.706	0.8950

*These data are based on a previously reported study [23]

### IL-based VA-MSPD approach coupled with Qubit detection

IL-based VA-MSPD was developed to directly incorporate the DNA enriched IL- and MIL-cosolvent mixtures in the qPCR assay where the DNA would be thermally desorbed into the qPCR buffer. An additional purification step was not required as DNA isolated by the method from the majority of plant species was of sufficient quality and quantity for qPCR, demonstrating that it can be applied to amplification-based techniques. However, fluorometric detection techniques such as Qubit are currently incompatible with the thermal desorption of DNA directly into the buffer and hence requires an additional DNA recovery step. Extractions were carried out with 1.5 mg of treated *C. pepo* plant tissue (as described earlier) and DNA from the resulting plant extract was separated and recovered from the plant matrix with silica spin columns (Nucleospin Plant II), according to the manufacturer’s protocol, using 60 µL of IL-DMSO-water mixture containing plant DNA as an input. The final elution step was performed with 50 µL of elution buffer containing Tris-HCl. As shown in Fig. S12, the DNA mass determined by both qPCR and Qubit was within error (*p* > 0.05) suggesting that the VA-MSPD approach can be coupled with Qubit detection through the incorporation of an additional purification step. Similarly, IL-based extractions were conducted on an additional 9 plants with 40–60 µL of the resulting IL-DMSO-water extracts undergoing spin column purification. The selection of these 10 plant species aimed to ensure diversity by avoiding duplication within higher order taxa. The effect of plant mass on DNA extracted was also tested by using 10 mg of plant tissue (data not shown); however, an improvement in the DNA mass was not observed likely due to the dilution of DNA with the corresponding increase in volumes required of the extraction solvents. Table 3 provides a concise overview of the outcomes and efficacy of the extraction and amplification results for 10 plant species examined in the study.

**Table 3:** Summary of DNA extraction efficiency, amplification success with *rbcL* and ITS markers and DNA quality assessment using the IL-based VA-MSPD approach

	Assessment of DNA quality						Plant matrix effect on qPCR	Assessment of DNA yield
	ITS marker			rbcL marker			BRAF	
Plant species	Amplification success with Cq values (*n* = 3)	Melt peaks of PCR product	Nonspecific bands on agarose gel	Amplification success with Cq values (*n* = 3)	Melt peaks of PCR product	Nonspecific bands on agarose gel	Amplification of non-target DNA	Mass of extracted DNA (ng/mg of plant tissue)
1. *Magnolia soulangeana* Soul.-Bod.	33.14 ± 1.72 (delayed)	Double peaks	Not tested	22.18 ± 0.10 (successful)	Single peak	Single band	No matrix effect	14.08 ± 4.07
2. *Nicotiana tabacum* L.	27.16 ± 0.60 (successful)	Single peak	Single band	25.91 ± 0.47 (successful)	Single peak	Single band	No matrix effect	15.02 ± 1.06
3. *Viburnum opulus* L.	no amplification	-	-	22.94 ± 0.59 (successful)	Single peak	Single band	Slight matrix effect	2.49 ± 0.29
4. *Cladrastis kentukea* (Dum. Cours.) Rudd	33.24 ± 0.50 (delayed)	Single peak	Not tested	22.24 ± 0.46 (successful)	Single peak	Single band	No matrix effect	6.62 ± 2.09
5. *Cucurbita pepo* L.	24.44 ± 0.31 (successful)	Single peak	Single band	23.05 ± 0.89 (successful)	Single peak	Single band	No matrix effect	7.56 ± 3.69
6. *Aesculus glabra* Willd.	24.96 ± 0.22 (successful)	Single peak	Single band	19.23 ± 0.25 (successful)	Single peak	Single band	No matrix effect	13.00 ± 7.73
7. *Tilia americana* L.	21.71 ± 0.27 (successful)	Single peak	Single band	20.08 ± 1.06 (successful)	Single peak	Single band	No matrix effect	4.78 ± 0.40
8. *Dieffenbachia* ‘Tropic Snow’	34.68 ± 1.20 (delayed)	Single peak	Non-specific bands	30.22 ± 0.75 (delayed)	Single peak	Single band	No matrix effect	5.01 ± 1.25
9. *Lilium henryi* Baker	34.26 ± 1.24 (delayed)	Inconsistent peaks	Non-specific bands	31.51 ± 0.45 (delayed)	Single peak	Single band	No matrix effect	6.84 ± 0.96
10. *Pennisetum glaucum* R. Br.	Cq value not determined (delayed)	-	-	19.94 ± 0.26 (successful)	Single peak	Single band	No matrix effect	29.49 ± 0.43

The overall performance of the IL-based VA-MSPD approach was evaluated against the NucleoSpin Plant II commercial kit in terms of DNA yield using both fresh and ethanol-pretreated tissue of *Arabidopsis thaliana*. Despite significant differences in the sample amounts and chemicals used, the extraction processes and processing time between the two methods, DNA yields were normalized to the mass of sample used. As detailed in Table S3, the commercial kit yielded a higher DNA mass per milligram of pretreated tissue, while the IL-based VA-MSPD method was more effective for fresh tissue, producing a greater DNA mass per milligram.

## Discussion

The present study demonstrates the broad scope of the miniaturized IL-based VA-MSPD approach across 17 plant species belonging to 13 different plant families, representing a broad range of diversity. All plants examined in this study are angiosperms and included 13 dicots and 4 monocots. Selection of the plant species for this study was intentional to target a wide diversity of plants with different plant metabolite chemistries, leaf anatomies, and defensive compounds to deter predation, in an effort to examine the versatility and broad application of the method across the plant Tree of Life. Selection of the [P_66614_^+^] [NTf_2_^−^] IL and [P_66614_^+^] [Ni(hfacac)_3_^−^ ] MIL as extraction solvents is based on previous studies where they have been used to successfully extract DNA from plant tissues and proven to be compatible with qPCR [15, 22, 23]. 

Beyond its utility in DNA barcoding, the nrITS region is frequently chosen as an ideal locus from the nuclear genome for species-level plant molecular phylogenetics due to its biparental inheritance, universality, and simplicity [26, 35]. Among the 17 plant species tested, successful qPCR amplification for ITS region was achieved only for 7 plant species, whereas 8 plant species demonstrated delayed amplification and 2 plant species completely inhibited the reaction. qPCR tests performed by spiking in non-target 98 bp DNA template and amplifying with the IL-based plant extract demonstrated that the co-extracted components from the plant matrix is either negligible or do not interfere with the enzymatic reaction for the majority of plant species with few exceptions such as *Q. macrocarpa* which exhibited complete inhibition, *V. opulus* and *K. paniculata* which demonstrated slightly delayed amplification. *Q. macrocarpa* (an oak) is known to be a challenging plant genus for DNA extraction due to the presence of high levels of phenolic substances and secondary metabolites that are difficult to eliminate [36, 37]. Inhibition of DNA amplification for *Q. macrocarpa* is likely due to either the co-extraction of polyphenolics and polysaccharides which can bind with DNA making it inaccessible to the polymerase enzyme or secondary metabolites that inhibit enzymatic activity [38–40]. Ethanol treatment can be a viable option in tissue preservation and removal of chlorophyll and secondary metabolites however, it may not be the ideal pretreatment method for plant taxa such as oaks. Delayed or no amplification from ITS for the majority of plant species may be due to inefficient or inconsistent amplification. Although ITS is one of several plant DNA barcode loci and has higher discriminatory power for comparative phylogenetics, it is known to suffer from non-specific amplification and lower success in PCR and sequencing [7]. Although a number of primer sets are available that target the ITS region, amplification and sequencing this region can be difficult [10]. Therefore, to improve the reliability in amplification, an additional marker, *rbcL* was tested.

It has been reported that the use of plastid genome has been more accessible compared to the nuclear genome and could potentially provide advantages for plant barcoding [26]. The plastid *rbcL* barcoding marker can be easily amplified, sequenced, and aligned in many land plants, serving as a valuable foundation for barcoding, even though its discriminatory power is somewhat limited [10]. Successful DNA amplification of *rbcL* for the majority of plant species in this study indicates that this method can be applied to many dicots, as well as some families of monocots. This study also demonstrated that the DNA isolated by the approach using IL and MIL offer greater amplification success with *rbcL* compared to that of ITS. Failure of certain markers to amplify DNA in some plant species may not be directly attributable to the DNA extraction method itself nor to the inherent quality of the DNA obtained. Instead, these failures may be related to factors such as primer specificity or the presence of secondary metabolites that interfere with the amplification process. These outcomes highlight the biological variability among different species and the complexities involved in DNA extraction and amplification from different plant species. Nevertheless, both nuclear and plastid DNA can be extracted by the approach. Future studies will seek to refine this protocol by exploring alternative amplification strategies, such as the use of different markers and the inclusion of additional steps or reagents that can help mitigate the effects of PCR inhibitors commonly found in plant extracts.

The MIL extracts of almost all the tested plant species demonstrated successful amplification with the control BRAF DNA template except for C. kentuckea indicating the possibility of inhibitory components being extracted. Furthermore, extended preservation of DNA within IL- and MIL-cosolvent mixtures was successfully demonstrated through qPCR amplification of the DNA-enriched extracts stored for 21 days at room temperature. C. kentuckea was an exception which demonstrated decreased fluorescence intensities in the amplification curves for both IL-DMSO-water and MIL-DMSO extracts upon storage. This may be due to the effect of inhibitory components which can interfere with the fluorescent qPCR assay. These results indicate that DNA extracted by this approach can be stored at room temperature for a time period up to 3 weeks prior to analysis.

It is also worth highlighting that DNA extraction from the herbarium sample resulted in a DNA mass comparable to that of a fresh sample indicating that the technique is capable of recovering DNA from highly degraded plant materials even after an extended period of storage. However, the efficacy of a method for DNA extraction from herbarium specimens also relies on the conditions to which specimens are exposed during both sampling and storage, and this efficiency might vary among different taxonomic groups [25, 41, 42]. Therefore, further studies are needed to evaluate the robustness of the method for ancient plant specimens from different plant taxa and collections that have been preserved under different conditions. The compatibility of the developed method for quantitative analysis was evaluated using qPCR and Qubit dsDNA high sensitivity assay demonstrating comparable results. Nevertheless, Qubit measurements necessitate an additional purification step unlike qPCR, due to the compatibility of the extraction solvents.

Plant DNA barcodes remain a highly efficient and robust tool for specialists and non-specialists alike to identify unknown plant samples to the correct genus, family, and even sometimes species. One of the objectives of the study was to demonstrate that the developed method yielded DNA of satisfactory quality for sequencing of DNA extracted from representative dicot and monocot species, and it has been accomplished successfully (data not shown). The search outcomes revealed top matches for either the exact species or the same genus of a number of closely related species demonstrating that the DNA extracted by this novel method not only successfully amplified DNA from each sample, but also the extracted DNA was able to be used for downstream Sanger sequencing studies.

The IL-based VA-MSPD method is distinguished by its miniaturized process, simplicity and minimal time requirement for the extraction [23]. Although certain chemicals involved in the synthesis of the IL and MIL extraction solvents, such as trihexyl(tetradecyl)phosphonium chloride, lithium bis[(trifluoromethane)sulfonyl]imide, and 1,1,1,5,5,5-hexafluoroacetylacetone may be acutely toxic, the extraction solvents themselves do not exhibit these toxic properties [20]. Additionally, the quantities used in the approach are minimal, especially when compared to the volumes of hazardous solvents typically employed in traditional phenol-chloroform extraction techniques.

One limitation of the study is the absence of fragment size analysis to determine the integrity of the DNA extracted. Maximizing the size of isolated DNA fragments is a complex challenge influenced by a variety of factors, in addition to the isolation method itself. Large DNA fragments, crucial for long-read sequencing technologies (e.g., PacBio), are prone to rapid degradation over time. The integrity of these fragments can be affected by numerous other factors, including the amount of time since death (or tissue removal from living organism), the temperature the sample was preserved in, the preservation method, etc [43]. To thoroughly assess the influence of the isolation method on fragment size, it would be beneficial to implement a more robust experimental design that accounts for more of these variables using a high number of samples and replicates for each variable and compared with the widely used DNA extraction protocols.

## Conclusions

This study successfully demonstrated the robustness of the IL-based VA-MSPD approach in lysing and extracting DNA from milligram fragments of plant tissues from diverse families across both dicots and monocots. In contrast to conventional methods that incorporate time-consuming procedures, the current technique facilitates plant DNA extraction with minimal sample and solvents while avoiding extended incubation steps significantly reducing the overall sample preparation time. The compatibility of the method with downstream applications such as qPCR, Qubit and Sanger sequencing without an additional purification step prior to amplification highlights its efficiency. Although *rbcL* demonstrated greater amplification success in the majority of plant species, amplification of both *rbcL* and nuclear ribosomal ITS barcoding regions validated the success of the approach in extracting plastid and nuclear DNA respectively. Extracted DNA in IL- and MIL-DMSO mixtures demonstrated stability at room temperature up to 3 weeks. Application of the method to an herbarium specimen dating back a century underscored its versatility. Future studies should expand the scope of genomic coverage to include high-throughput sequencing techniques and whole genome sequencing to explore the utility of extracted DNA for increasingly modern and next generation molecular applications that aim to recover whole genome sequences and/or expand the amount of sequenced genomic loci for enhanced species discrimination. We envision this approach will be a valuable tool in the toolkit of biologists and policymakers who require efficient and scalable techniques for downstream applications in molecular biology, such as agriculture, conservation, ecology, evolution, forensics, and more.

## Methods

### Chemicals and materials

Nickel (II) chloride (98%), ammonium hydroxide (28–30% solution in water) 1,1,1,5,5,5-hexafluoroacetylacetone (99%) and glycerol (≤ 99%) were purchased from Acros Organics (Morris Plains, NJ, USA). Ethanol (200 proof) and silver nitrate (AgNO_3_, ≥ 99.9%) were purchased from MilliporeSigma (St. Louis, MO, USA). Trihexyl(tetradecyl)phosphonium chloride (97.7%) was purchased from Strem Chemicals (Newburyport, MA, USA). Methanol (99.7%) and lithium bis[(trifluoromethane)sulfonyl]imide ([Li^+^] [NTf_2_^−^]) were purchased from Sigma Aldrich (St. Louis, MO, USA). Agarose (genetic analysis grade), dimethyl sulfoxide (DMSO) (≥ 99.7%), optically clear PCR caps and tube strips were acquired from Thermo Fisher Scientific (Waltham, MA, USA). Anhydrous diethyl ether (99.0%) was acquired from Avantor Performance Materials Inc. (Center Valley, PA, USA). All primers shown in Table S1 were purchased from Integrated DNA Technologies (Coralville, IA, USA). SYBR Green I (10,000x) was purchased from Life Technologies (Carlsbad, CA, USA). A NucleoSpin Plant II commercial kit (Macherey–Nagel, Düren, Germany) was purchased from Fisher Scientific. A 50 bp DNA ladder was purchased from Gold Biotechnology (St Louis, MO, USA). A QIAquick Gel Extraction Kit was purchased from QIAgen (Valencia, CA, USA). Agarose gel electrophoresis was carried out using a Bethesda Research Laboratories H4 Horizontal Gel Electrophoresis system (Life Technologies) and a dual output power supply (Neo/Sci, Rochester, NY, USA). A Milli-Q water purification system (Bedford, MA, USA) was used to supply 18.2 MΩ•cm deionized water for the preparation of aqueous solutions. An Elechomes UH401 food dehydrator (Elechomes, China) was used for removal of residual solvent in the leaf dehydration experiments. An Eppendorf I24 incubator shaker (Eppendorf, Hamburg, Germany) was used as an incubator for extraction experiments. An agate mortar (50 mm O.D. x 43 mm I.D. x 12 mm depth) with a pestle acquired from MSE supplies (Tucson, AZ, USA) was used for extraction experiments.

### MIL and IL synthesis

Synthesis and characterization of the [P_66614_^+^] [NTf_2_^−^] IL and [P_66614_^+^] [Ni(hfacac)_3_^−^] MIL used in this study was carried out based on previously reported procedures [20, 44]. Their chemical structures are shown in Fig. S13.

### Plant Specimen collection and sample pretreatment

Leaf samples from 17 different plant species belonging to 13 different families (Table 1) were collected from field sampling in Ames, Iowa. For all samples collected, herbarium vouchers were deposited at ISC, the Ada Hayden herbarium (Ames, Iowa). The herbarium specimen of *Cucurbita pepo* L. (accession no. 96,352) was obtained from ISC. Fresh leaf fragments weighing approximately 100 mg were immersed in 10 mL of ethanol at 37 ^◦^C in an incubator for 15 h. A 10 mL volume of fresh ethanol was added after 12 h for samples from which chlorophyll was not completely leached out. Residual solvent in the leaves was removed using a food dehydrator at 35 ^◦^C for 3 h until a constant mass was reached. The mass loss upon sample pretreatment was recorded for each plant sample (Fig. S1). A similar procedure was carried out for the herbarium sample. To evaluate the impact of the sample pretreatment in ethanol on plant DNA extraction by the IL-based VA-MSPD approach, control extraction experiments were carried out for 1.5 mg of *Arabidopsis thaliana* plant tissue that had undergone ethanol dehydration for both 0.5 h and 12 h, as well as for air-dried plant tissue without any ethanol pretreatment.

### DNA standard preparation and qPCR amplification

For the preparation of DNA standard solutions, genomic DNA was isolated using a NucleoSpin Plant II commercial kit (Macherey–Nagel, Düren, Germany) following the manufacturer’s specifications and the concentration of each extract was determined by fluorometric detection using a Qubit 4.0 fluorometer (ThermoFisher Scientific, Waltham, MA, USA) with the 1X- double-stranded DNA (dsDNA) high sensitivity assay.

Plant DNA extracted by the IL and MIL was used as the template for qPCR amplification. Part of the nuclear internal transcriber spacer (ITS) region of the plant genome was amplified by qPCR using the ITS-3 and ITS-4 universal primer set [45]. All reactions were performed using a Bio-Rad CFX96 Touch Real-time PCR thermocycler (Hercules, CA, USA) with a total volume of 20 µL. Each reaction containing either 0.5 µL of the DNA enriched IL-DMSO-Water or MIL-DMSO mixture required the following components: 1 × SsoAdvanced Universal SYBR Green Supermix, 200 nM of each ITS primer and additional 1 × SYBR green I. The thermocycling conditions were as follows: initial denaturation step of 10 min at 95 ^◦^C and 40 cycles comprised of a 15 s denaturation step at 95 ^◦^C and a 45 s annealing step at 65 ^◦^C, followed by an optical detection step. Melt curve analysis was carried out after qPCR amplification and began at 65 ^◦^C for 5 s while increasing to 95 ^◦^C in 0.5 ^◦^C increments.

A partial *rbcL* sequence was amplified by qPCR using the *rbcLa*-F and *rbcLa*-R primer set [46]. All reactions were performed in a total volume of 20 µL. Each reaction containing either 0.5 µL of the DNA enriched IL-DMSO-Water or MIL-DMSO mixture required the following: 1 × SsoAdvanced Universal SYBR Green Supermix, 600 nM of each *rbcLa* primer and an additional 0.5 × SYBR green I. The thermocycling conditions were as follows: initial denaturation of 10 min at 95 ^◦^C, 40 cycles of a 30 s denaturation step at 95 ^◦^C, a 30 s annealing step at 55 ^◦^C and 1 min extension step at 72 ^◦^C, followed by an optical detection step. Melt curve analysis was carried out after qPCR amplification starting at 65 ^◦^C for 5 s and increasing to 95 ^◦^C in 0.5 ^◦^C increments. The cycle of quantification (Cq) values obtained by the qPCR experiments were used to assess the amount of amplifiable DNA. Calibration curves were constructed by plotting the Cq values against the log of mass of DNA per reaction. All qPCR experiments were carried out in triplicate.

Amplification of spiked BRAF template DNA (98 bp DNA sequence of the BRAF gene) sequence with 0.5 µL of the DNA enriched IL-DMSO-Water or MIL-DMSO plant extract in the reaction required 1 × SsoAdvanced Universal SYBR Green Supermix, 1 µM BRAF primers and an additional 1 × SYBR green I. The thermocycling conditions included an initial denaturation of 2 min at 95 ^◦^C and 40 cycles of a 5 s denaturation step at 95 ^◦^C, followed by a 30 s annealing step at 60 ^◦^C and an optical detection step after each cycle. All custom-designed PCR assays are summarized in Table S2.

### Agarose gel electrophoresis conditions

To determine the integrity of the *rbcL* and ITS amplicons obtained by the amplification of genomic DNA extracted by the IL and MIL, agarose gel electrophoresis was performed. A 5 µL volume of 10% glycerol was added to 20 µL of the PCR product, mixed well and 20 µL of the sample was loaded on a 1% (w/v) agarose gel prepared with 1X Tris-acetate-EDTA (TAE) buffer along with a 50 bp DNA ladder. All gels were run for 1.5 h at 70 V and the bands visualized using a Safe Imager 2.0 transilluminator (Invitrogen, Carlsbad, CA, USA).

### IL/MIL-based vortex assisted matrix solid phase dispersion (VA-MSPD) approach for extraction of plant DNA

A previously developed IL-based VA-MSPD approach for the model plant, *A. thaliana*, was used in this study [23]. Briefly, pretreated plant tissue weighing 1.5 ± 0.2 mg was transferred into an agate mortar and 15 µL of the IL was added and dispersed followed by the addition of 30 µL DMSO. After homogenizing the sample, the plant-IL-DMSO mixture was transferred into a qPCR tube followed by the addition of 15 µL water. The mixture was vortexed for 30 s and centrifuged for 30 s at 13,000 × g. The same procedure was followed for the MIL-based extraction using an optimized volume of 1:4 (v/v) for MIL: DMSO. A 0.5 µL aliquot of the supernatant was used for qPCR analysis. IL-based extractions were carried out for 17 plant species and 1 herbarium sample while MIL-based extractions were carried out for 10 plant species.

### Electronic supplementary material

Below is the link to the electronic supplementary material.

Supplementary Material 1

## Data Availability

No datasets were generated or analysed during the current study.
