# Supplementary material for: Versatile DNA extraction from diverse plant taxa using ionic liquids and magnetic ionic liquids: a methodological breakthrough for enhanced sample utility
Source: Plant Methods. 2024 Jun 14;20:91. doi: 10.1186/s13007-024-01217-z (PMC11177442; doi:10.1186/s13007-024-01217-z)
Supplement: Supplementary file 1 — Supplementary Material 1 [file 13007_2024_1217_MOESM1_ESM.pdf]

Electronic Supplemental Information

Versatile DNA extraction from diverse plant taxa using ionic liquids and magnetic ionic liquids: A methodological breakthrough for enhanced sample utility.

Shashini De Silva, Cecilia Cagliero, Morgan R. Gostel, Gabriel Johnson, and Jared L. Anderson*

Author details

SD: Department of Chemistry, Iowa State University, Ames, Iowa 50011, USA, email: shashini@iastate.edu

CC: Dipartimento di Scienza e Tecnologia del Farmaco, Università di Torino, I-10125, Turin, Italy, email: cecilia.cagliero@unito.it

MRG: Botanical Research Institute of Texas, Fort Worth, Texas 76107-3400, USA, email: mgostel@brit.org

GJ: Smithsonian Institution, Suitland, Maryland 20746, USA, email: JohnsonG@si.edu

JLA: Department of Chemistry, Iowa State University, Ames, Iowa 50011, USA, email: andersoj@iastate.edu

* Corresponding author:

Jared L. Anderson

Department of Chemistry

Iowa State University

Ames, IA 50011

Tel.: +1 515-294-8356

E-mail address: andersoj@iastate.edu

Table of Contents

Table S1	p. S3
Table S2	p. S4
Table S3	p. S5
Fig. S1	p. S6
Fig. S2	p. S7
Fig. S3	p. S8
Fig. S4	p. S9
Fig. S5	p. S10
Fig. S6	p. S11
Fig. S7	p. S12
Fig. S8	p. S13
Fig. S9	p. S14
Fig. S10	p. S15
Fig. S11	p. S16
Fig. S12	p. S17
Fig. S13	p. S18

Table S1: Sequences of the primers used in this study.

Name	Sequence
rbcLa Forward	5′-ATGTCACCACAAACAGAGACTAAAGC-3′
rbcLa Reverse	5′-GTAAAATCAAGTCCACCRCG-3′
ITS 3	5’-GCA TCG ATG AAG AAC GCA GC-3’
ITS 4	5’-TCC TCC GCT TAT TGA TAT GC-3’
BRAF Forward	5’-TTCATGAAGACCTCACAGTAAA-3’
BRAF Reverse	5’-GGATCCAGACAACTGTTCAA-3’

Table S2. Summary of PCR assays used in this study.

DNA Sequence	IL-cosolvent or MIL-cosolvent mixture composition	IL-cosolvent or MIL-cosolvent mixture volume (µL)	Reaction Volume (µL)	Buffer Type	Primers used	Primer Concentration (nM)	Additives
Plant Genomic DNA	1:2:1 (v/v/v) IL:DMSO:water mixture or 1:4 (v/v) MIL:DMSO	0.5	20	SSO Supermix	ITS3 and ITS4	200	1x SYBR Green
Plant Genomic DNA	1:2:1 (v/v/v) IL:DMSO:water mixture or 1:4 (v/v) MIL:DMSO	0.5	20	SSO Supermix	*rbcLa*_F and *rbcLa*_R	600	0.5x SYBR Green
BRAF DNA	1:2:1 (v/v/v) IL:DMSO:water mixture or 1:4 (v/v) MIL:DMSO	0.5	20	SSO Supermix	BRAF forward and BRAF reverse	1000	1x SYBR Green

Table S3. Comparison of performance of IL-based VA-MSPD method with NucleoSpin Plant II commercial kit for *Arabidopsis thaliana* plant tissue.

**Tissue type**	**Ethanol pre-treated**		**Fresh**	
**Extraction method**	**NucleoSpin Plant II commercial kit**	**IL-based VA-MSPD method**	**NucleoSpin Plant II commercial kit**	**IL-based VA-MSPD method**
**Sample amount (mg)**	20 ± 0.2	1.5 ± 0.2	100 ± 0.3	26.5 ± 0.3
**DNA mass (ng/mg of plant tissue)**	7.65 ± 0.17	2.31 ± 0.14	2.01 ± 0.09	19.79 ± 1.99

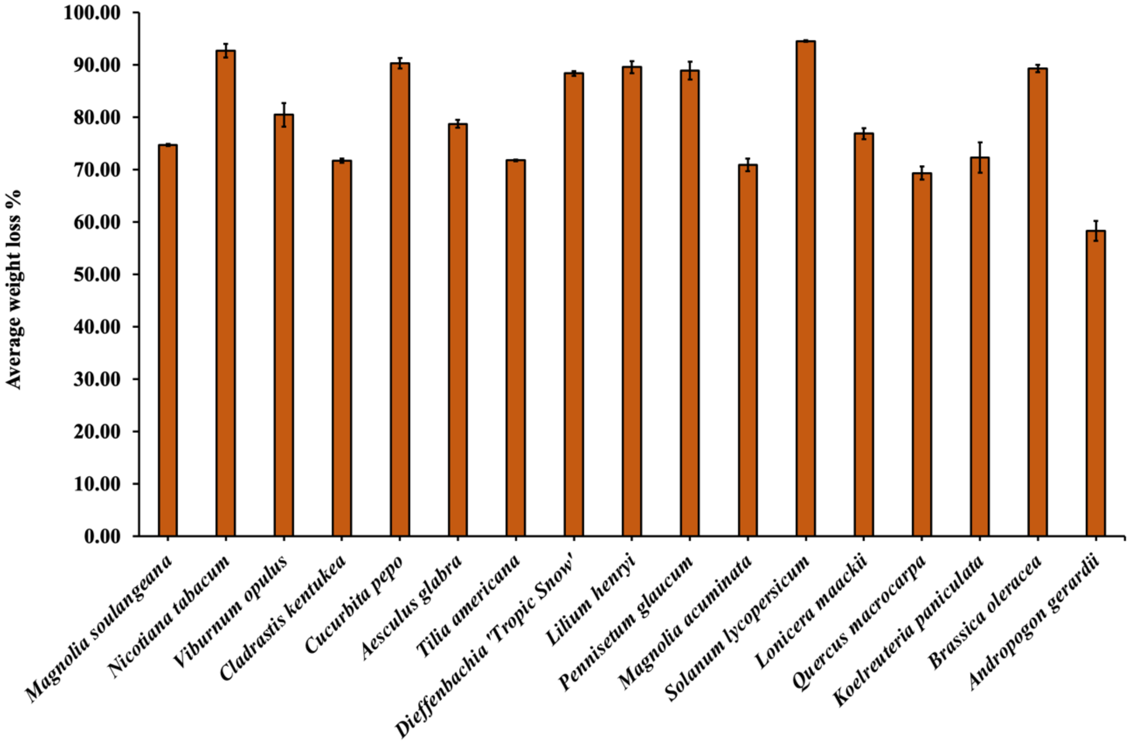

Fig. S1. Weight loss observed for fresh leaves when immersed in absolute ethanol for 15 h at 37 °C in an incubator followed by removal of residual solvent in a food dehydrator at 35 °C for 3 h. All experiments were conducted in triplicate.

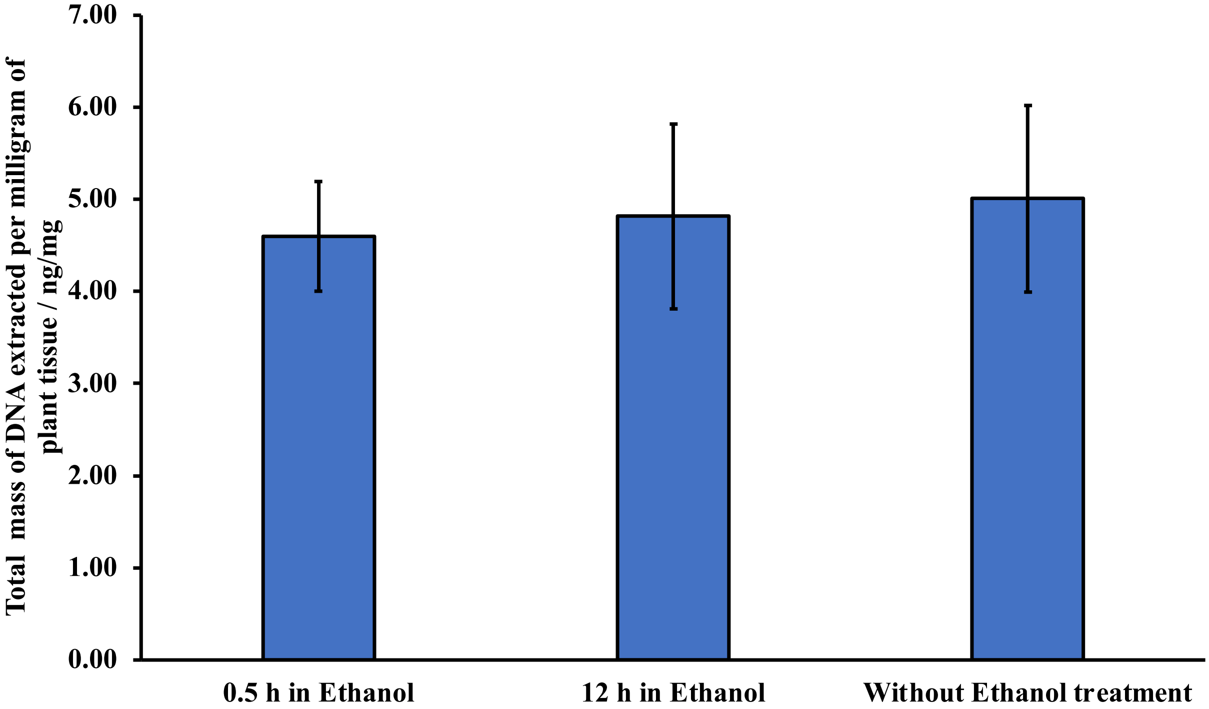

Fig. S2. The effect of the sample pretreatment by ethanol on plant DNA extraction by the IL-based VA-MSPD approach. Extractions were performed using 1.5 mg of *Arabidopsis thaliana* plant tissue employing 15 µL of $[P_{6,6,6,14}^{+}][NTf_2^{-}]$ IL.

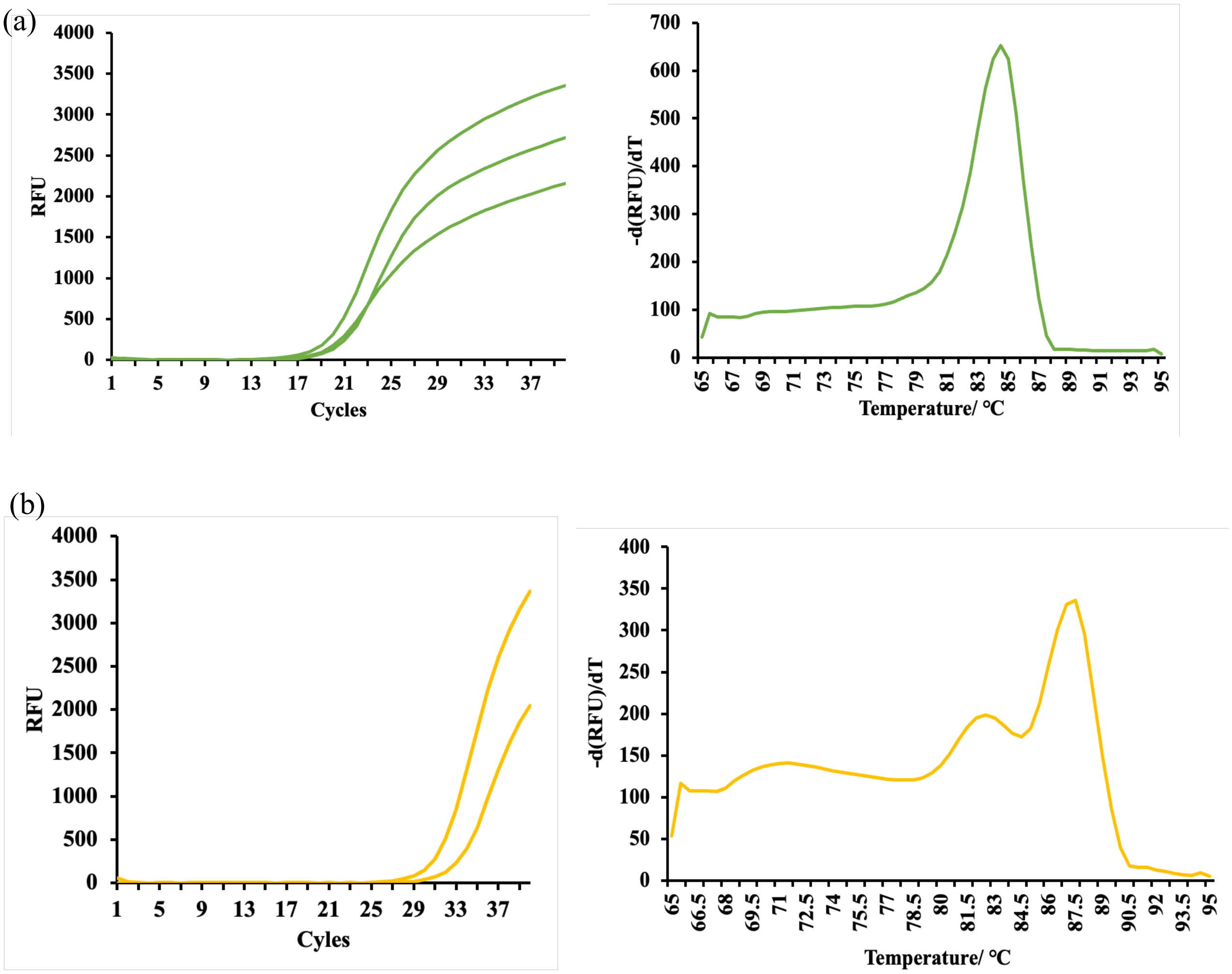

Fig. S3. Amplification curves and melt peaks obtained by qPCR amplification of (a) *rbcL* and (b) ITS marker for *Magnolia soulangeana* genomic DNA extracted by the VA-MSPD approach employing 15 μL of [$P_{6,6,6,14}^{+}$][NTf_2^{-}] IL.

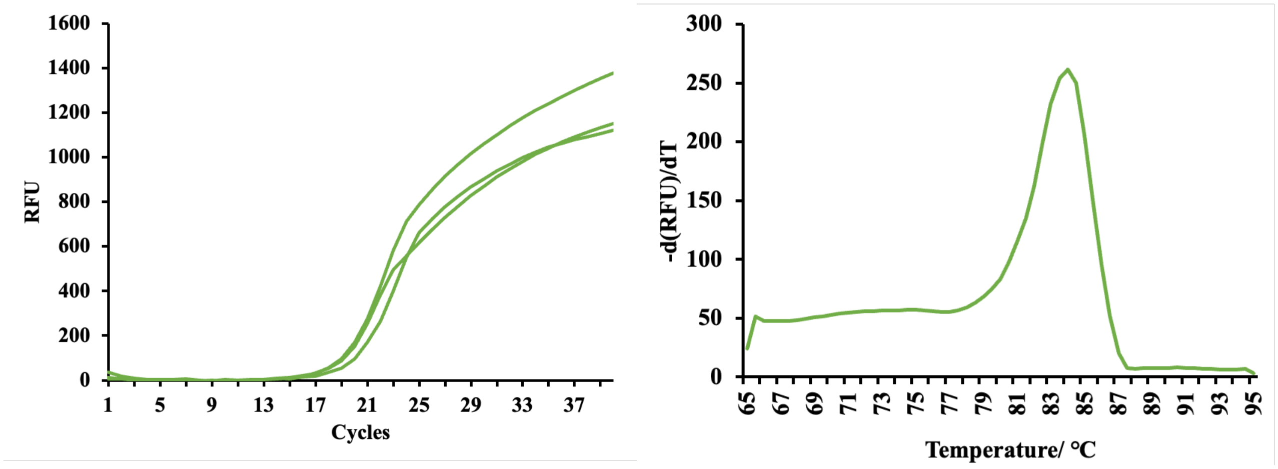

Fig. S4. Amplification curves and melt peaks obtained by qPCR amplification of *rbcL* marker for *Viburnum opulus* genomic DNA extracted by the VA-MSPD approach employing 15 μL of $[P_{6,6,6,14}^{+}][NTf_2^{-}]$ IL. No amplification was observed for ITS marker. All experiments were conducted in triplicate.

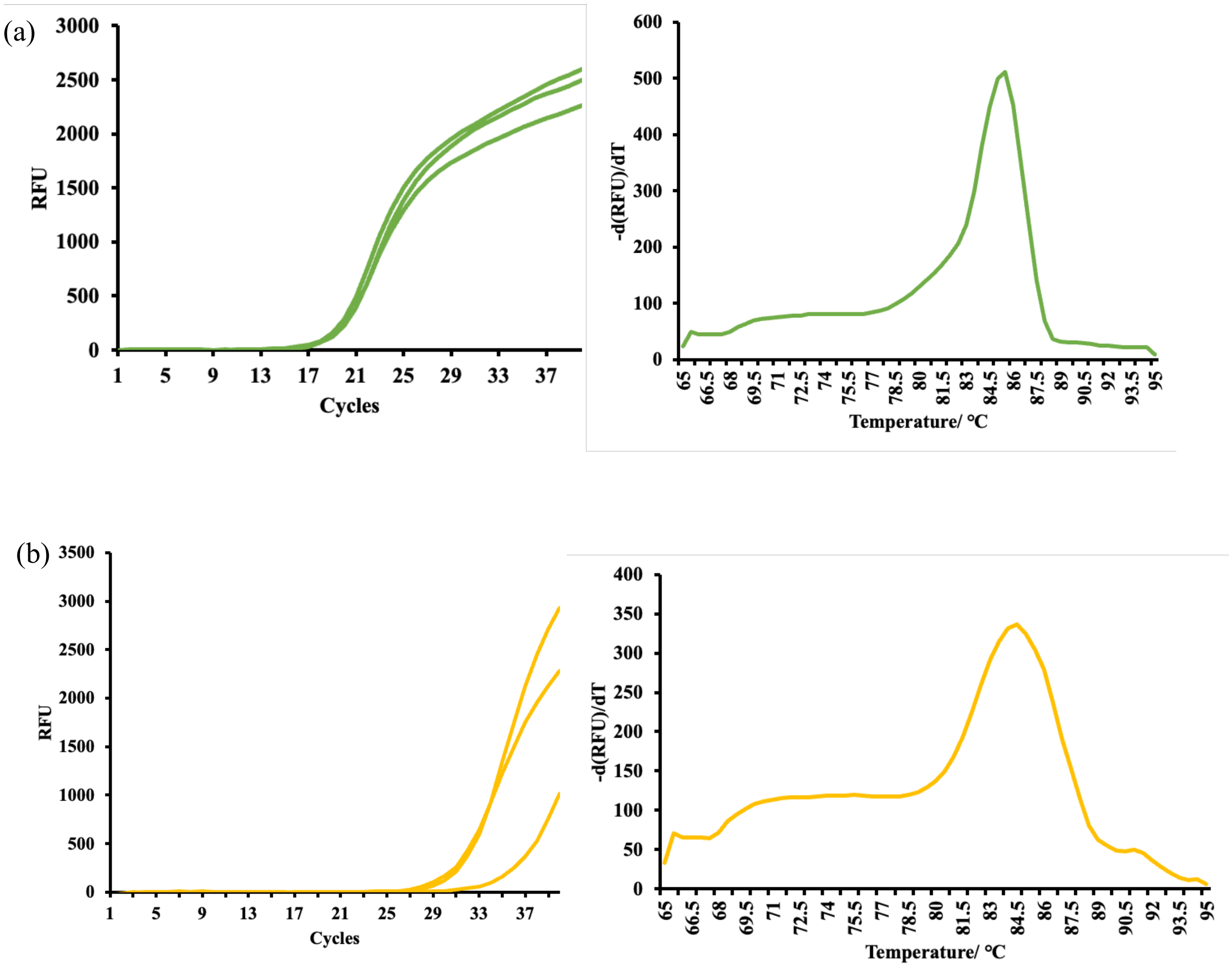

Fig. S5. Amplification curves and melt peaks obtained by qPCR amplification of (a) *rbcL* and (b) ITS marker for *Lonicera maackii* genomic DNA extracted by the VA-MSPD approach employing 15 µL of [$P_{6,6,6,14}^{+}$][NTf_2^{-}] IL. All experiments were conducted in triplicate.

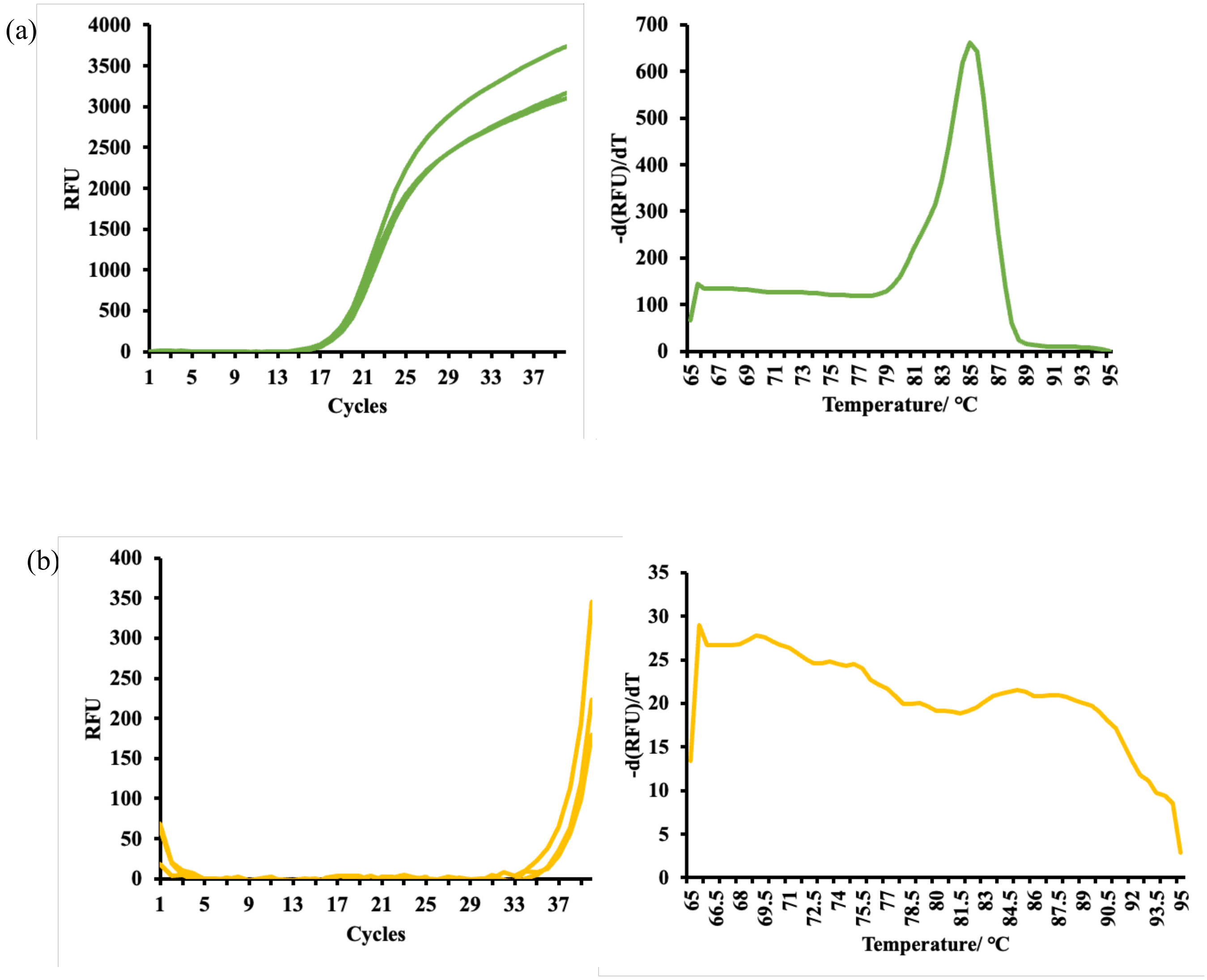

Fig. S6. Amplification curves and melt peaks obtained by qPCR amplification of (a) *rbcL* and (b) ITS marker for *Pennisetum glaucum* genomic DNA extracted by the VA-MSPD approach employing 15 µL of $[P_{6,6,6,14}^{+}][NTf_2^{-}]$ IL. All experiments were conducted in triplicate.

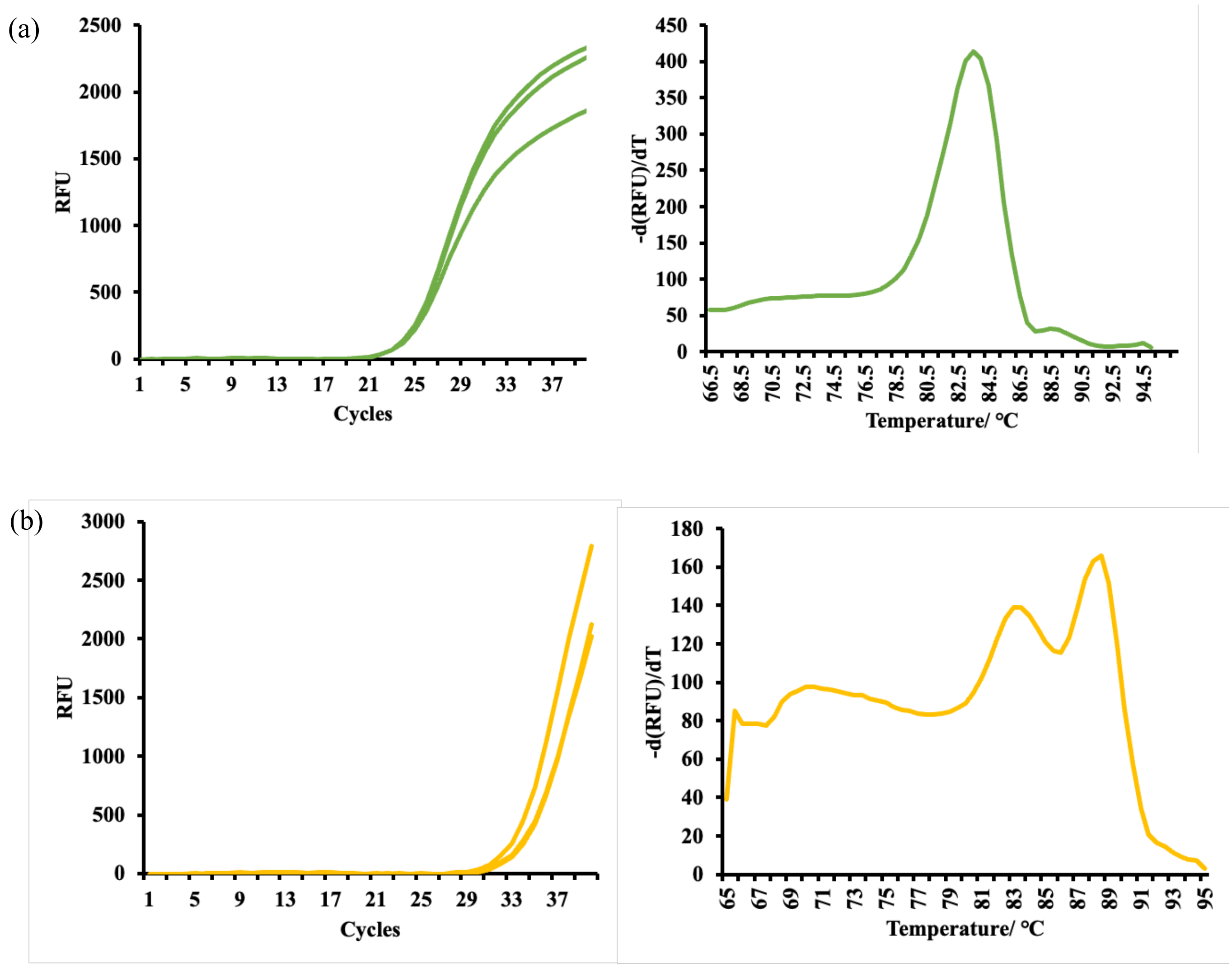

Fig S7. Amplification curves and melt peaks obtained by qPCR amplification of (a) *rbcL* and (b) ITS marker for *Andropogon gerardii* genomic DNA extracted by the VA-MSPD approach employing 15 µL of $[P_{6,6,6,14}^{+}][NTf_2^{-}]$ IL. All experiments were conducted in triplicate.

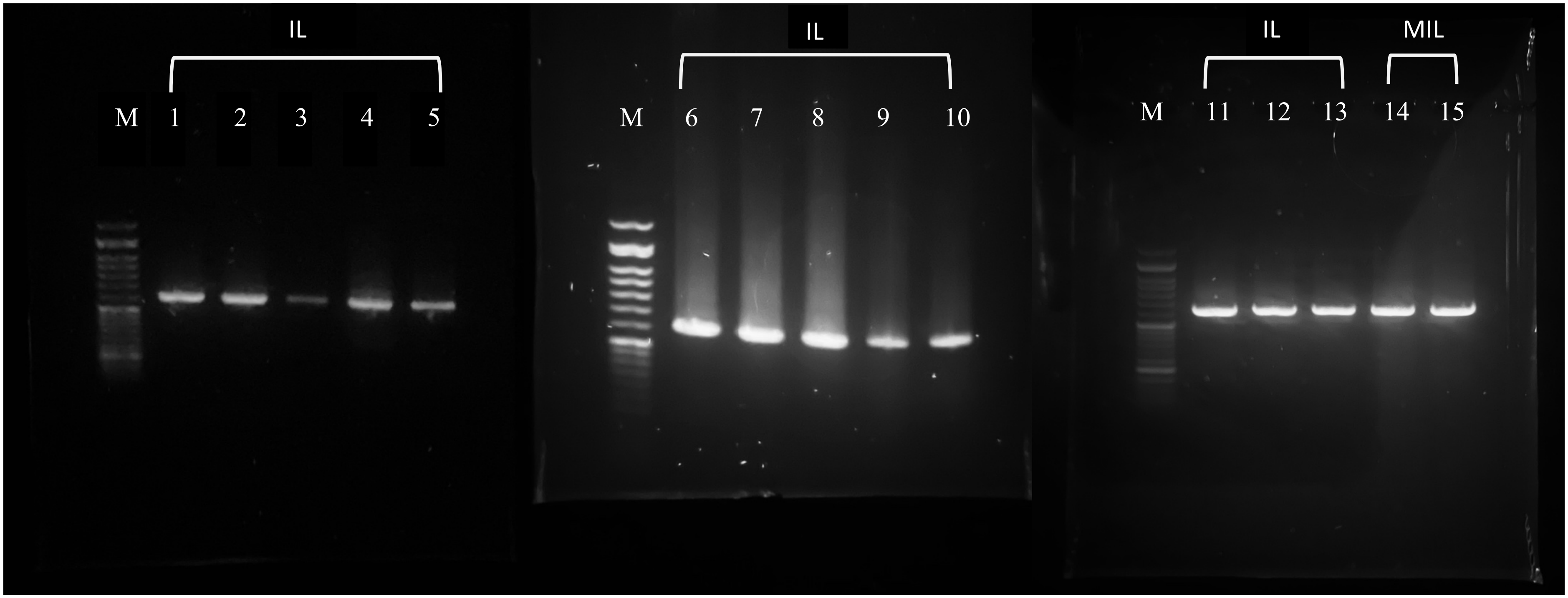

Fig. S8. PCR products derived from the amplification of plant DNA extracted using the IL-/MIL-based VA-MSPD approach. The *rbcL* PCR products are shown for: 1: *M. soulengeana*, 2: *V. opulus*, 3: *Cladrastis kentuckea*, 4: *Cucurbita pepo*, 5: *Nicotiana tabacum*, 6: *L. maackii*, 7: *Solanum lycopersicum*, 8: *Brassica oleracea*, 9: *Dieffenbachia* 'Tropic Snow', 10: *Lilium henryi*, 11: *Aesculus glabra*, 12: *Tilia americana*, 13: *Koelreuteria paniculata* 14: *A. glabra* 15: *T. americana* M: 50 bp molecular size marker

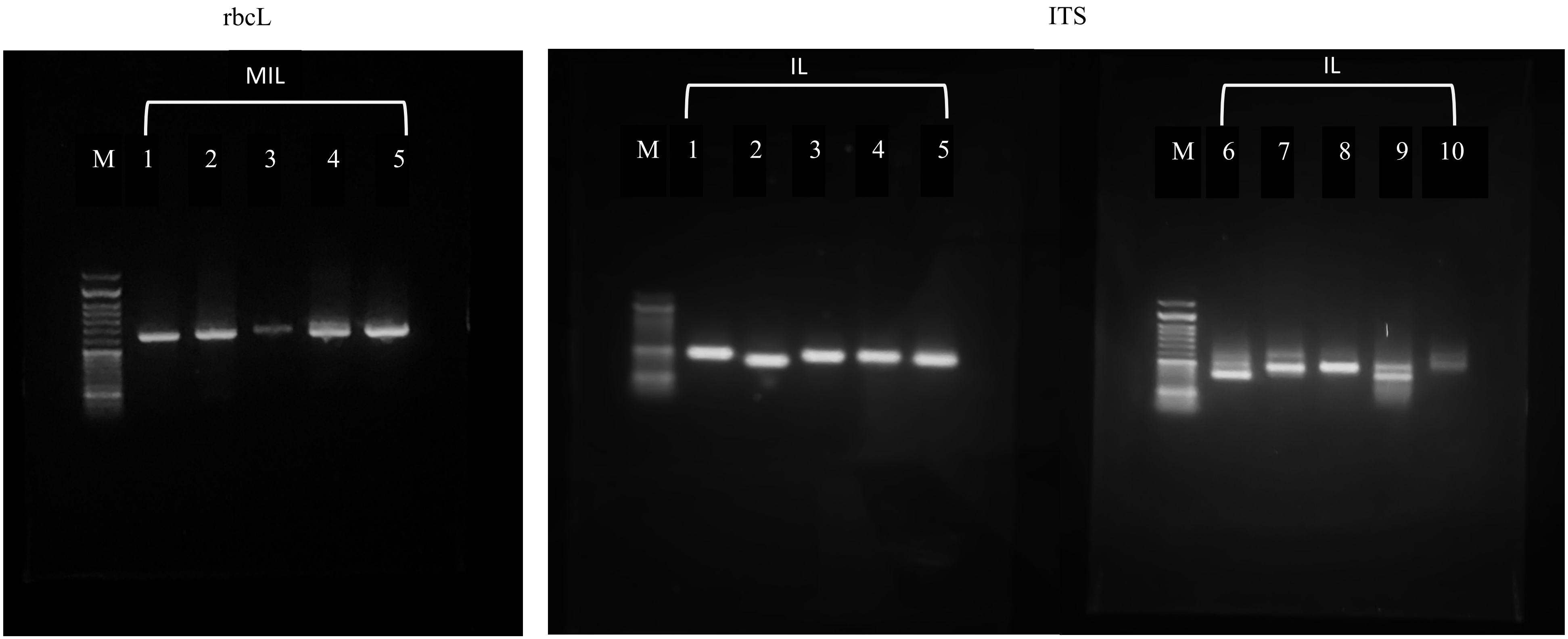

Fig. S9. PCR products derived from the amplification of plant DNA extracted using the IL-/MIL-based VA-MSPD approach. The *rbcL* PCR products are shown for: 1: *M. soulengeana*, 2: *N. tabacum* 3: *C. kentuckea*, 4: *C. pepo*, 5: *V. opulus* and ITS PCR products for 1: *C. pepo* 2: *B. oleracea* 3: *K. paniculata* 4: *T. americana* 5: *N. tabacum* 6: *S. lycopersicum* 7: *A. gerardii* 8: *A. glabra* 9: *Dieffenbachia* 'Tropic Snow' 10: *L. henryi* M: 50 bp molecular size marker

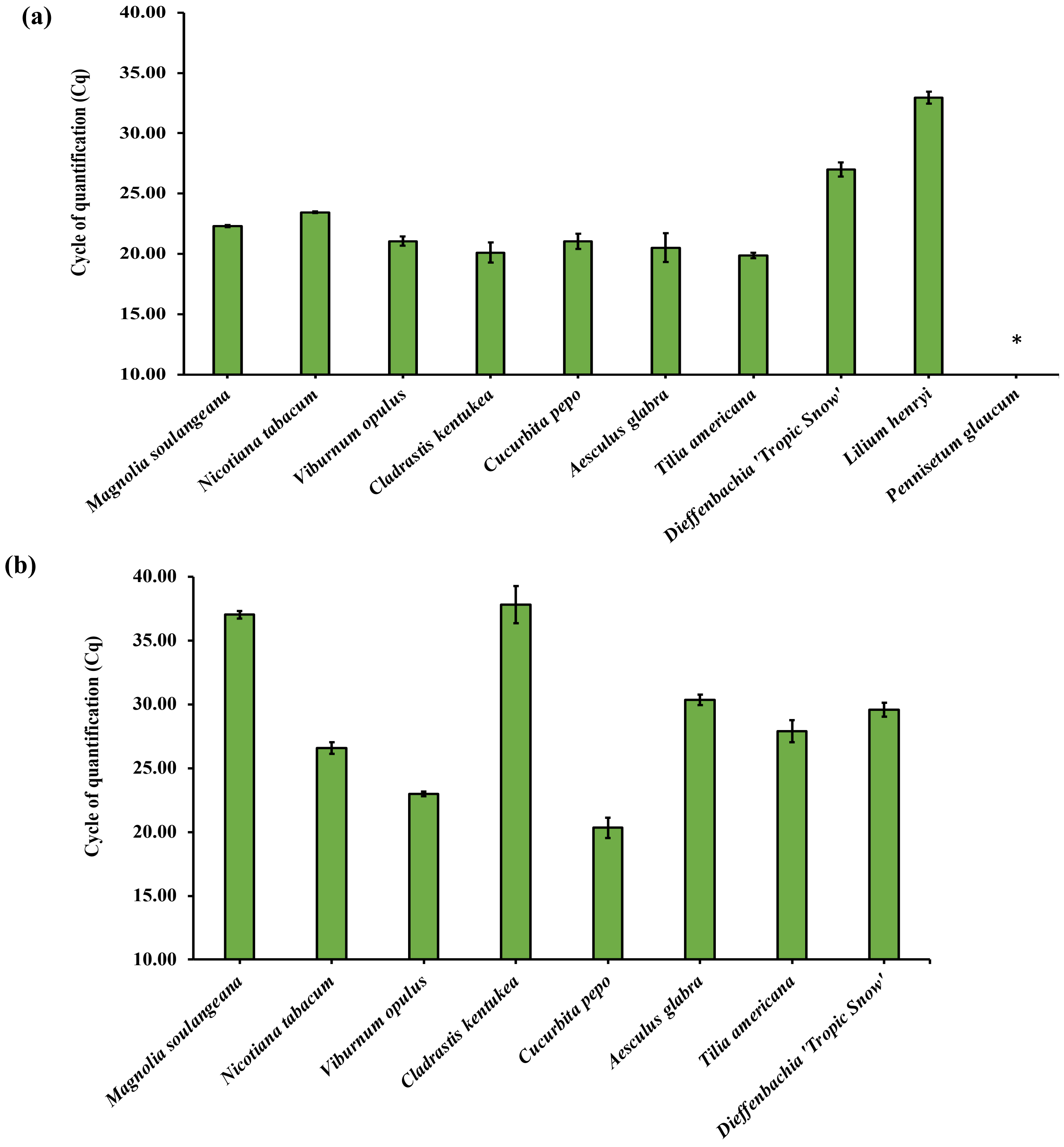

Fig. S10: Cq values as a measure of amplification success for the (a) *rbcL* marker and (b) ITS marker derived from qPCR amplification of plant DNA extracted by the MIL-VA-MSPD procedure employing 1.5 mg of treated plant tissue and 15 μL of $[P_{6,6,6,14}^{+}][Ni(hfacac)_3^{-}]$ MIL. Extractions were carried out in triplicate. (Cq>30 is considered as delayed amplification) Note: *No amplification was observed.

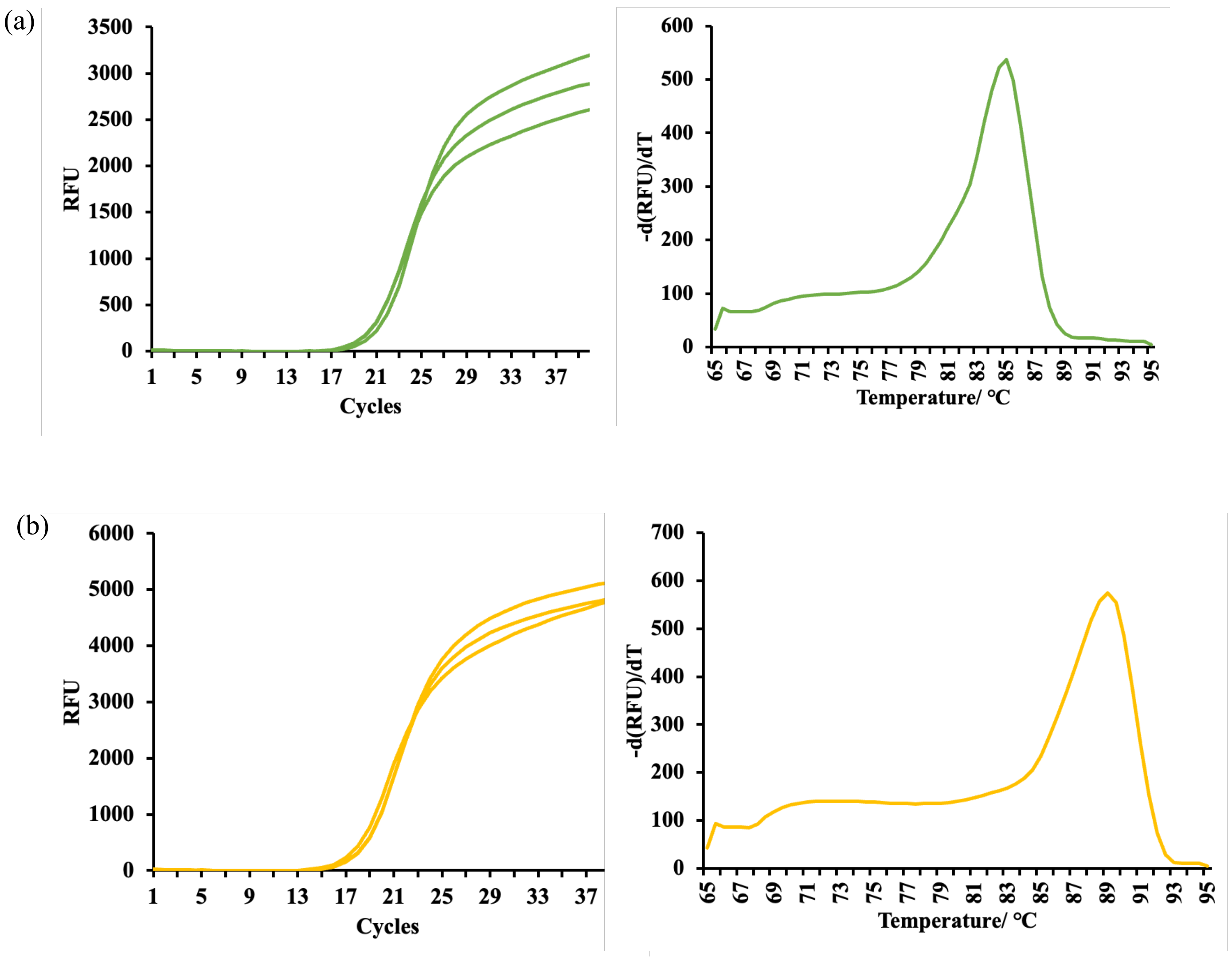

Fig. S11: Amplification curves and melt peaks obtained by qPCR amplification of (a) *rbcL* and (b) ITS marker for genomic DNA extracted from 1.5 mg of *C. pepo* herbarium specimen by the VA-MSPD approach employing 15 μL of $[P_{6,6,6,14}^{+}][NTf_2^{-}]$ IL.

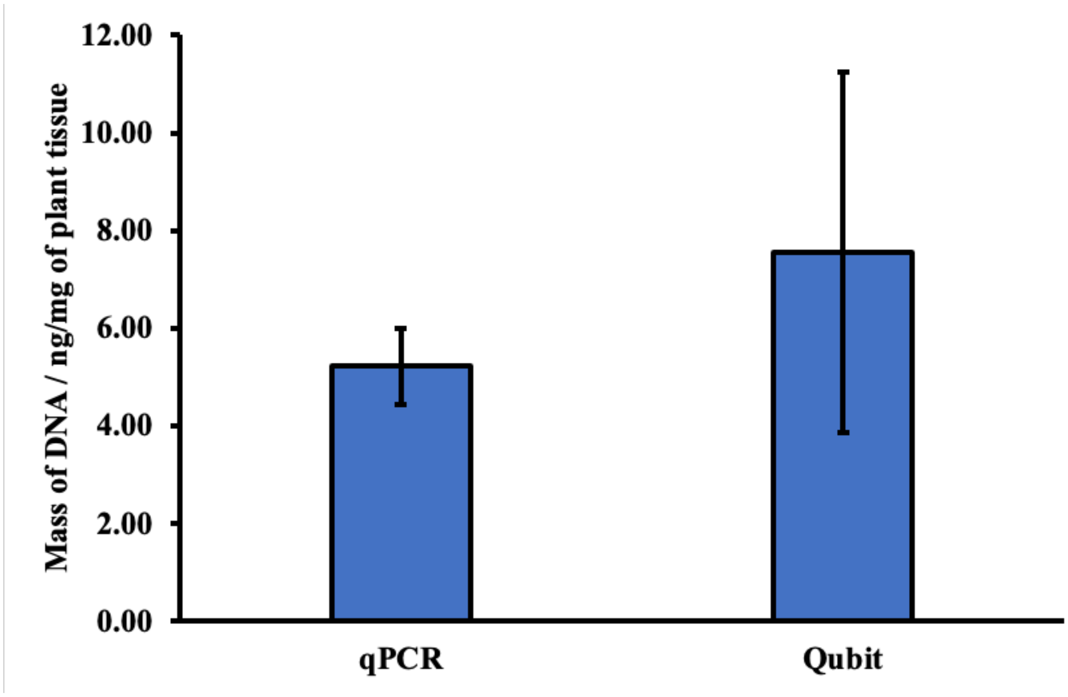

Fig. S12: Comparison of the amount of DNA recovered from the $[P_{6,6,6,14}^{+}][[NTf_2^{-}]$ IL-DMSO-water mixture and quantified using qPCR and Qubit. Triplicate extractions were carried out using the VA-MSPD approach employing 1.5 mg of plant tissue and 15 μL of $[P_{6,6,6,14}^{+}][NTf_2^{-}]$ IL.

(a)

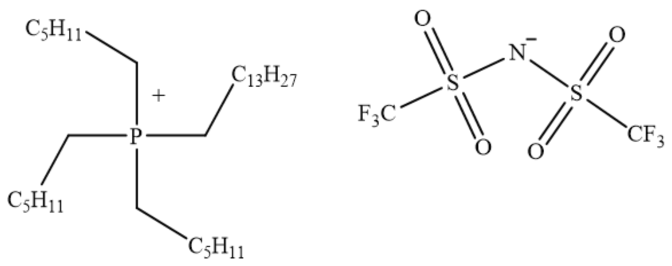

(b)

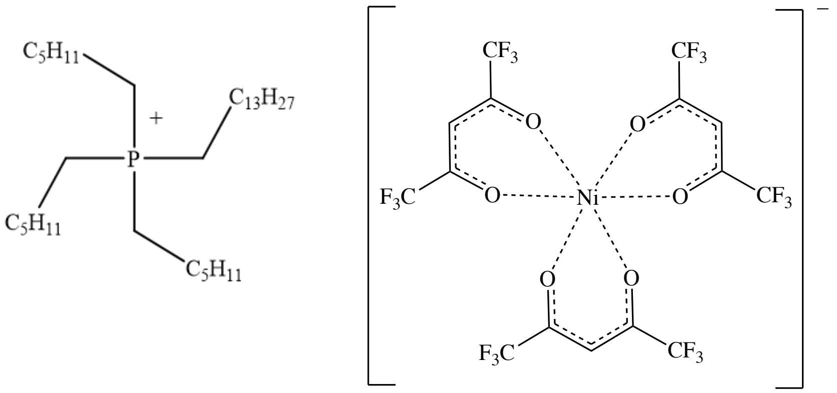

Fig. S13. Structures of the hydrophobic IL and MIL examined in this study. (a) $[P_{66614}^{+}][NTf_2^{-}]$ IL and (b) $[P_{66614}^{+}][Ni(hfacac)_3^{-}]$ MIL.
